# LITE microscopy: Tilted light-sheet excitation of model organisms offers high resolution and low photobleaching

**DOI:** 10.1083/jcb.201710087

**Published:** 2018-05-07

**Authors:** Tanner C. Fadero, Therese M. Gerbich, Kishan Rana, Aussie Suzuki, Matthew DiSalvo, Kristina N. Schaefer, Jennifer K. Heppert, Thomas C. Boothby, Bob Goldstein, Mark Peifer, Nancy L. Allbritton, Amy S. Gladfelter, Amy S. Maddox, Paul S. Maddox

**Affiliations:** 1 Department of Biology, The University of North Carolina at Chapel Hill, Chapel Hill, NC; 2 Department of Chemistry, The University of North Carolina at Chapel Hill, Chapel Hill, NC; 3 Joint Department of Biomedical Engineering, The University of North Carolina at Chapel Hill and North Carolina State University, Chapel Hill and Raleigh, NC

## Abstract

Fluorescence microscopy is a powerful approach for studying subcellular dynamics at high spatiotemporal resolution; however, conventional fluorescence microscopy techniques are light-intensive and introduce unnecessary photodamage. Light-sheet fluorescence microscopy (LSFM) mitigates these problems by selectively illuminating the focal plane of the detection objective by using orthogonal excitation. Orthogonal excitation requires geometries that physically limit the detection objective numerical aperture (NA), thereby limiting both light-gathering efficiency (brightness) and native spatial resolution. We present a novel live-cell LSFM method, lateral interference tilted excitation (LITE), in which a tilted light sheet illuminates the detection objective focal plane without a sterically limiting illumination scheme. LITE is thus compatible with any detection objective, including oil immersion, without an upper NA limit. LITE combines the low photodamage of LSFM with high resolution, high brightness, and coverslip-based objectives. We demonstrate the utility of LITE for imaging animal, fungal, and plant model organisms over many hours at high spatiotemporal resolution.

## Abstract (precis)

Fadero et al. present lateral interference tilted excitation (LITE) microscopy–a tilted light-sheet method to illuminate high-numerical-aperture objectives for fluorescence microscopy. LITE can be implemented unobtrusively on most microscope systems and combines low photodamage with high resolution and efficient detection in imaging fluorescent organisms.

## Introduction

**Figure 1.**
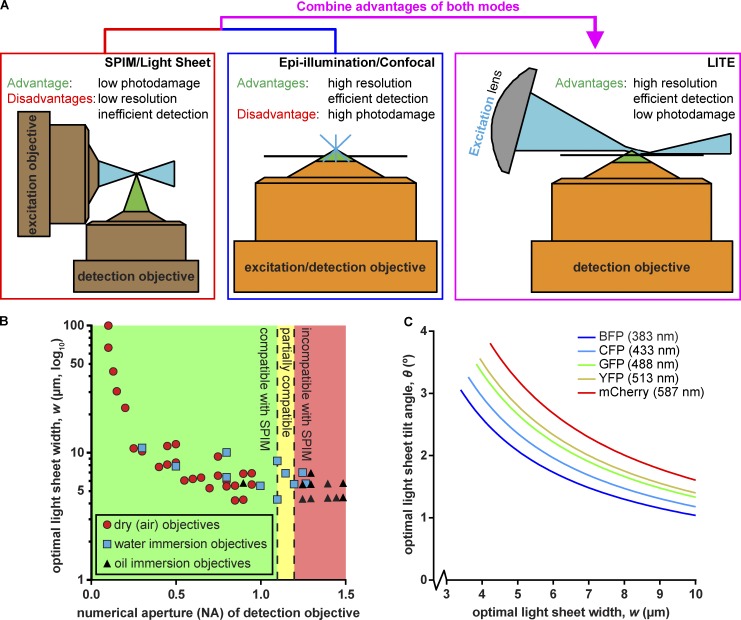
**Rationale and theory behind LITE. (A)** We combined low photodamage of SPIM/LSFM (left) with high-NA objectives (orange) of epi-illumination/confocal microscopy (center) to create LITE (right). LITE tilts a cylindrical lens (gray) to focus a laser (blue) into a sheet onto the coverslip surface (black line). **(B)** Scatterplot of calculated optimal light-sheet width for LITE, based on Eq. 7, for 90 commercially available objectives (plotted by increasing NA). Green area: objectives that can/have been used with existing live-cell SPIM/LSFM technologies. Yellow area: objectives that have been used with live-cell SPIM/LSFM by means of unconventional geometries. Red area: objectives previously incompatible with live-cell SPIM/LSFM. **(C)** Theoretical optimal light sheet width, *w*, as a function of optimal sheet tilt angle, θ. Ideal light sheet parameters are traced for five common fluorescent proteins: blue fluorescent protein (BFP), cyan fluorescent protein (CFP), green fluorescent protein (GFP), yellow fluorescent protein (YFP), and monomeric Cherry (mCherry). Wavelengths of excitation light plotted in C correspond to maximal-absorption wavelength of each protein.

To properly visualize and measure cellular and subcellular dynamics, cell biologists demand imaging at high spatial and temporal resolution. The fluorescence microscope is a popular, modern tool used to address these demands and solve cellular dynamics problems. However, conventional fluorescence microscope modalities require high-intensity light to illuminate the sample through the objective lens, exciting all fluorophores in the path of the collimated excitation light. The fluorophores emit light that is collected by the objective lens and transmitted to the detector. A disadvantage of the traditional “epi-illumination” geometry is that light is emitted from fluorophores outside the focal plane and contributes to the image, which confounds the focal information. Confocal microscopy mitigated this problem by selectively collecting light from the focal plane through the use of conjugate pinholes (Stelzer et al., 1995). However, the reduction of out-of-focus fluorescence by confocal microscopy does not overcome the need for high-intensity illumination light that generates out-of-focus excitation events (Fig. 1 A, blue box). High-intensity illumination transmits intense energy to the sample, damaging fluorophores that release reactive oxygen species upon photobleaching. Consequently, these reactive oxygen species chemically damage living samples through phototoxicity (Laissue et al., 2017). The most common method for reducing both out-of-focus excitation and emission, total internal reflection fluorescence microscopy, can only illuminate regions of the cell within ∼200 nm of the coverslip surface (Axelrod, 1981).

Light-sheet fluorescence microscopy (LSFM; or selective plane illumination microscopy [SPIM]) minimizes excitation-based photodamage by only partially illuminating the sample (Huisken et al., 2004). In the 15-yr existence of modern LSFM, various implementations have arisen, most of which use two traditional objective lens elements arranged orthogonally to illuminate the sample with a sheet of light and align the detection focal plane with the illuminating sheet (Huisken et al., 2004; Santi, 2011; Chen et al., 2014; Wu et al., 2016). LSFM reduces or eliminates out-of-focus excitation, increasing the signal-to-background ratio (SBR) for fluorophores in the focal plane (Fig. 1 A, red box). This higher SBR allows detection of image features with lower excitation energy, thus reducing the photodamage incurred with conventional optical configurations. These features allow the acquisition of a significantly larger number of exposures of a sample than any other mode of fluorescence microscopy. However, the orthogonal orientation of the illumination light sheet with respect to the detection objective generally requires that the sample be mounted at a minimum of 1 mm from the detection objective, forcing the use of low-NA (below 1.1) detection objective lenses. Therefore, the use of highly efficient, high-resolution, oil-immersion objectives is incompatible with current LSFM regimes (Fig. 1 A, red box).

The detection of subcellular structures that drive cell biological processes, including mitosis, endocytosis, and cytokinesis, require high-NA detection objectives because of their increased resolution and detection efficiency. Because 1.1 was the highest feasible NA detection objective (Chen et al., 2014) used with traditional geometries to view live cells (Fig. 1 B, green area), use of LSFM to study these subcellular structures with the traditional resolution or efficiency was not possible. Multiview SPIM geometries have been able to accommodate a 1.2-NA water-immersion objective (Wu et al., 2016) to increase the resolution and detection efficiency of LSFM (Fig. 1 B, yellow area); however, to approach the native resolution of oil-immersion objectives (Fig. 1 B, red area) traditionally used in cell biology, postacquisition deconvolution was required. This data processing has high requirements for time, user expertise, specialized software, and data storage, which are currently inaccessible to the average cell biology laboratory. Accordingly, there existed a need to build upon the currently available designs for LSFM by combining selective illumination with conventional microscope stands and objective lenses that enable detection and resolution of subcellular structures and dynamics.

Here we present lateral interference tilted excitation (LITE) microscopy, which we developed to use high-NA, oil-immersion objective lenses to image living, fluorescent samples illuminated by a light sheet (Fig. 1 A, magenta box). We achieved this goal by using a tilted sheet that can access the working distance of high-NA, oil- and water-immersion objective lenses, including a 60× 1.49-NA oil-immersion objective that accepts 88% more emitted fluorescence and offers a 26% increase in native lateral resolution compared with a 25× 1.1-NA water-dipping objective (Chen et al., 2014). LITE is compatible with traditional coverslip-based mounting conditions, meaning that LITE can be used with water- and oil-immersion objectives. The LITE method can also be implemented unobtrusively on most existing upright or inverted microscope systems, meaning high-resolution differential interference contrast or other microscopic modalities can be used simultaneously (or in rapid succession) with LITE imaging. LITE images do not require computational reconstruction to view; the native images received from the camera are the data. In sum, LITE microscopy combines the low photodamage of LSFM with the high-NA objective lenses to allow high spatiotemporal live-cell imaging.

## Results

### LITE illuminates a thin slice of fluorescent samples

**Figure 2.**
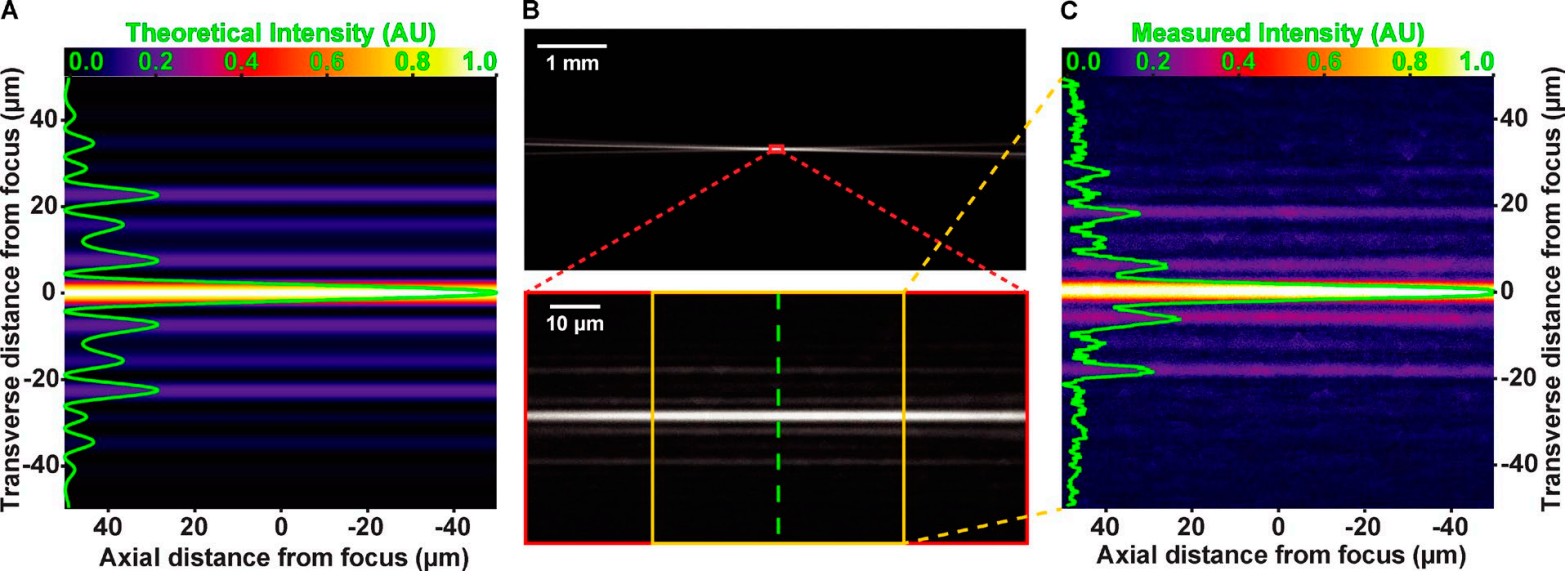
**Experimental verification of theoretical light sheet formation.** All images in show light sheet from the side. **(A)** Theoretical interference pattern at cylindrical lens focus. Image has been false-colored by “Fire” lookup table in Fiji (scale at top). Transverse (across sheet width) intensity line scan is overlaid in green. FWHM of the central peak is predicted to measure 4.3 µm. **(B)** Low-magnification image (upper) of light sheet focusing into fluorescent media. High-magnification image (lower) of cylindrical lens focal region. Green line indicates location of transverse line scan of measured intensity. **(C)** Subset of the image in lower portion of B (between the yellow lines). Line scan (green line), scale, and coloration are consistent with predicted pattern in A. Measured FWHM of the central sheet is 4.3 µm, in agreement with the theoretical prediction.

The feature shared by all SPIM/LSFM technologies is the spatial restriction of the illumination light to a volume on the order of magnitude of the detection objective’s focal plane, so that fluorophores outside of the focal plane do not experience unnecessary illumination. We used LITE microscopy to produce a sheet of light with constant thickness over the desired objective’s field of view (FOV; 150 µm). We theorized we could accomplish this thin illumination scheme using established cylindrical, lens-based cosine wave optics (Golub et al., 2015). We therefore calculated the theoretical side view of the light sheet to visualize the predicted sheet width and length (width = 4.3 µm, length = 270 µm; Fig. 2 A). To verify that our experimental light sheet recapitulates what our calculations predicted, we visualized the experimental sheet from the side at 1× magnification through a dilute solution of fluorescein (Fig. 2 B, top). We acquired a 40× magnified image of our experimental light sheet to quantify the width (Fig. 2 B, red box). When compared with the theoretical intensity profile (Fig. 2 A; Golub et al., 2015) predicted by the theoretical electric field amplitude at the focal plane of the masked cylindrical lens, our experimentally observed central peak had nearly identical sheet dimensions (*w* = 4.3 µm; *L****′*** = 296 µm; Fig. 2 D). Practically, we observed that excitation intensity was too low in these side lobes to generate signal in low-density fluorophore regimes, such as those of live cell imaging (unpublished data).

### LITE operates at native, diffraction-limited spatial resolution

**Figure 3.**
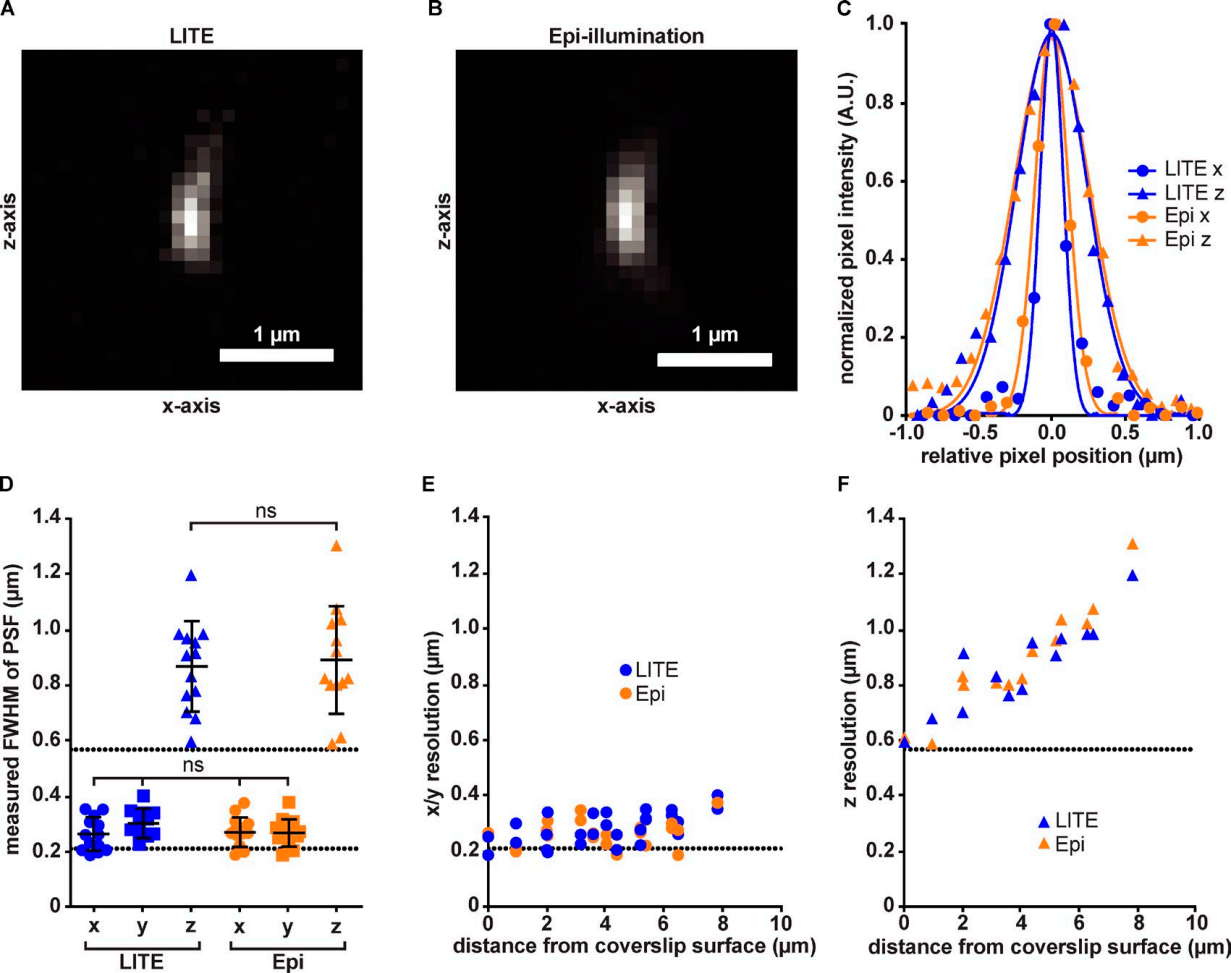
**Quantification of LITE spatial resolution. (A)** Image of a fluorescent 100-nm bead, visualized by using LITE. Image is maximal-intensity projected along y axis to show lateral (x) and axial (z) resolution. **(B)** Image of the same bead from A, visualized with epi-illumination. **(C)** Pixel intensity values for line scans across the x axis and z axis for LITE and epi-illumination images of the bead in A and B, respectively. Gaussian fits of intensity for each dimension are overlaid in corresponding colored lines. **(D)** Plots of FWHM for Gaussian fits to fluorescence intensity of all beads (*n* = 12) in x (circles), y (squares), and z (triangles) dimensions for LITE (blue) and epi-illumination (orange). Statistical significance assessed by Student’s *t* test (ns, P > 0.05). Error bars represent mean ± SEM. Upper and lower black dotted lines indicate theoretical axial and lateral (0.211 and 0.568 µm, respectively) resolution for this objective. **(E)** Scatterplot of each bead’s measured lateral (x/y) resolution as a function of the measured distance from the coverslip surface. Dotted line corresponds to predicted resolution from D. **(F)** Scatterplot of each bead’s measured axial (z) resolution as a function of measured distance from coverslip surface. Dotted line corresponds to predicted resolution from D.

The main goal of LITE microscopy is to combine the use of high-NA objectives to maximize resolution and detection efficiency with live-cell LSFM. We thus tested if LITE could be used with high-NA, oil-immersion objectives and provide the high resolution expected from those objectives. The spatial resolution of LITE images should depend solely on the objective NA and the wavelength of emitted fluorescent light. Therefore, spatial resolution in LITE images should be identical to spatial resolution in epi-illumination images, when the objective and samples are the same. To quantitatively test whether the spatial resolution is the same, we suspended subdiffraction (100-nm-diameter) fluorescent beads (Thermo Fisher Scientific) in 2% agarose and acquired images from the same field of beads using LITE (Fig. 3 A) and epi-illumination (Fig. 3 B) with a high-NA detection objective (60× 1.49-NA oil immersion). We then measured the point spread function of each bead in three dimensions by fitting a Gaussian trace to pixel intensity and interpolating the full width at half maximal intensity (FWHM) in each dimension (Fig. 3 C). The Gaussian FWHMs of beads visualized with LITE (blue) are identical to those visualized with epi-illumination (Fig. 3, D–F, orange). Spherical aberration artifacts in the z-resolution of the objective were similar for LITE and epi-illumination (Fig. 3 F), supporting the conclusion that LITE operates at the expected resolution for the chosen objective.

### LITE significantly reduces photobleaching compared with epi-illumination

As with other modalities of LSFM, the selective-plane illumination of LITE microscopy is expected to reduce the photodamage experienced by live fluorescent samples. To quantify the photobleaching rate of LITE-illuminated samples, we imaged early (1- to 4-cell) *Caenorhabditis elegans* embryos expressing fluorescently tagged (GFP) histone H2B (LP148 strain; Dickinson et al., 2013). To measure the true rate of GFP photobleaching without any confounding biological variables, such as new protein translation, proteolysis, and active transport of the fluorescent signal in the z-dimension, we needed a method to inhibit these biological processes. Accordingly, we immobilized the embryos by dissection into M9 nematode media plus 2 mM NaN_3_. This treatment inhibits ATP synthesis, thereby indirectly inhibiting ATP-dependent processes, such as protein translation, cytoskeleton motor protein activity, and proteolysis. Thus, any decrease in the measured fluorescent signal should be due to excitation-induced photobleaching.

**Figure 4.**
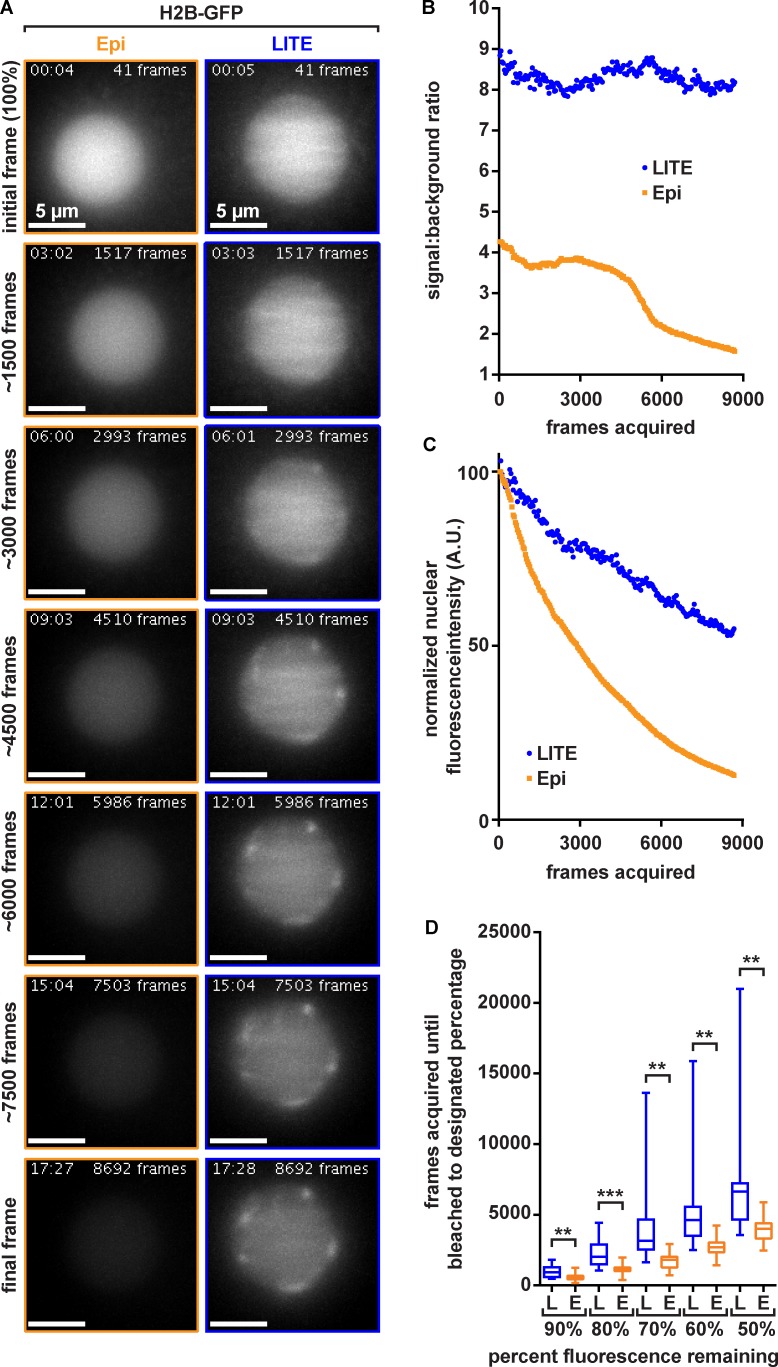
**Quantification of LITE photobleaching rates. (A)** Representative image sets of *C. elegans* embryos expressing GFP-tagged histone H2B construct to visualize nuclei. Representative images show P1 nucleus. All images were taken by using the same 60× 1.4-NA oil-immersion objective with a frame exposure time of 100 ms, a z-step size of 0.5 µm, a z range of 20 µm, and no delay between time points. Images shown are z maximal-intensity projections. Lengths of time the nuclei were exposed to the laser (LITE) or arc lamp (epi-illumination) are denoted in the upper left-hand corner of each image in the format of minutes:seconds. Cumulative number of frames acquired up until displayed images were acquired is denoted in the upper right-hand corner of each image. Rows represent images of the nuclei (internally scaled to initial frame of each nucleus) taken after the denoted number of frames (left of rows). **(B)** Measured SBRs of LITE and epi-illumination of nuclei shown in A. **(C)** The raw integrated density values of the nuclear regions-of-interest for LITE and epi image sets represented in A. **(D)** Box-and-whiskers plots of all nuclei (*n* ≥ 16), illustrating the number of frames acquired before nuclei bleached to 90, 80, 70, 60, and 50% intensities for both LITE (L; blue boxes) and epi-illumination (E; orange boxes). LITE significantly increases the number of frames that can be acquired before fluorescent nuclei bleach to 90, 80, 70, 60, and 50% of their original intensities (P < 0.01).

Fluorescent worm embryos were imaged under identical growth and mounting conditions using either epi-illumination or laser-illumination via LITE. The intensities of the epi-illumination field and the LITE laser were specified to generate images with similar initial starting characteristics: namely, SBR (qualitatively referred to as contrast) and raw integrated fluorescence density. We found that LITE preserves SBR over the course of imaging (Fig. 4, A and B). Epi-illumination (orange) starts at a lower SBR and approaches the lower limit of 1.0 (a level precluding analysis) more quickly than LITE (Fig. 4 B, blue).

In addition to preserving SBR, LITE also decreases the rate at which the fluorescent signal photobleaches. At equivalent frame numbers, the nucleus visualized with LITE is brighter than that visualized with epi-illumination (Fig. 4 C). We quantified the fluorescence intensities of nuclei over time and found that the detected fluorescence decreased more rapidly in the epi nucleus (orange) than in the LITE nucleus (Fig. 4 C, blue). To more thoroughly illustrate the photobleaching improvement from epi-illumination to LITE, we measured the number of frames we could acquire from nuclei before the samples bleached to 90, 80, 70, 60, or 50% (Fig. 4 D and Fig. S4). On average, LITE significantly increases the number of frames that can be acquired before the nuclei have bleached to a given percentage of starting intensity. In sum, compared with epi-illumination, LITE preserves SBR and reduces photobleaching.

### LITE is compatible with a variety of fluorescent organisms

**Figure 5.**
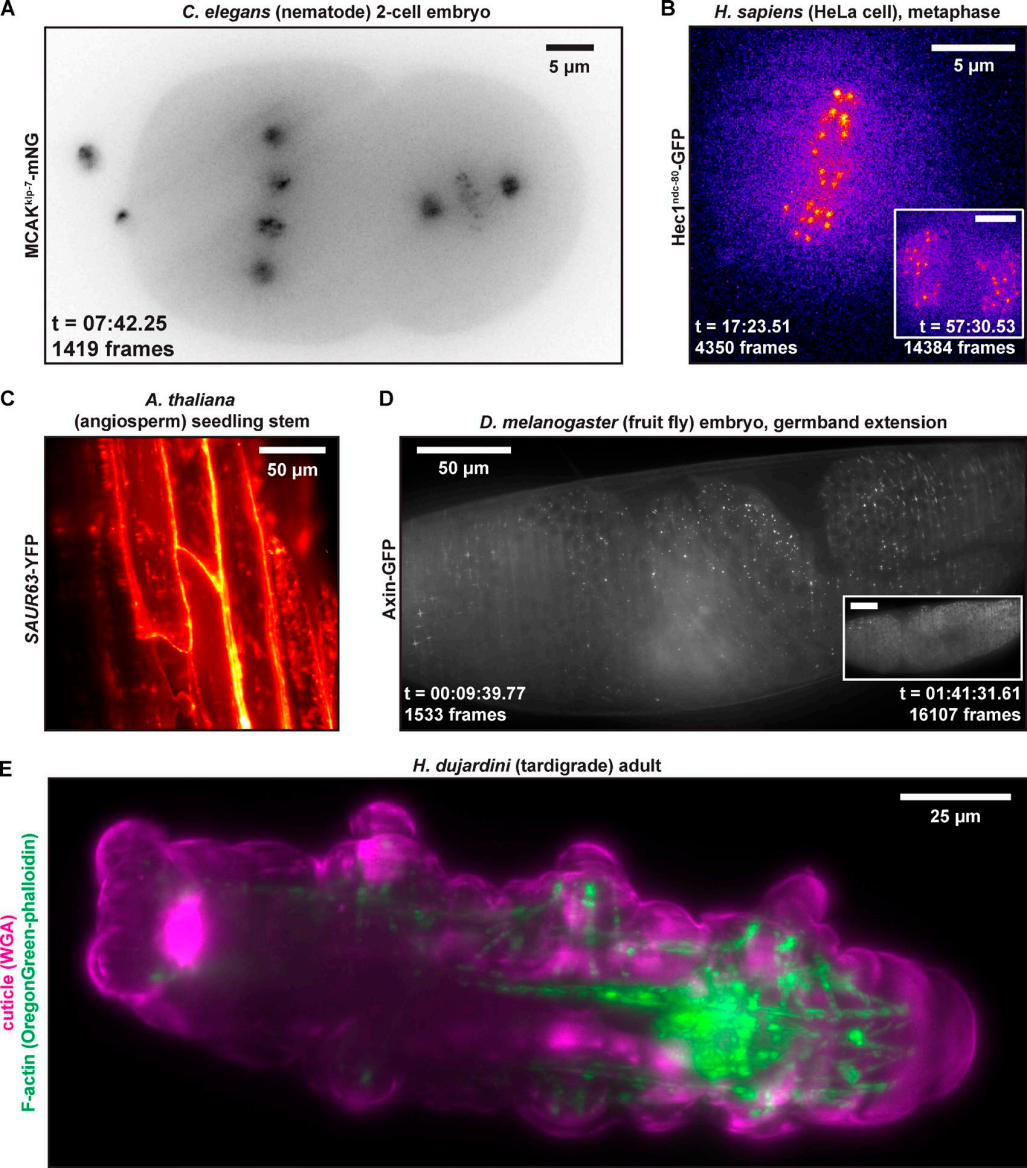
**Representative LITE fluorescent images taken of a variety of model organisms.** The organisms include *C. elegans* (A), *H. sapiens* (B), *A. thaliana* (C), *D. melanogaster* (D), and *H. dujardini* (E). Fluorescent constructs imaged in each organism are delineated to left of each representative image. Images presented in A, B, and D are taken from the full movies available in Videos 3, 6, and 4, respectively. Images in C and E are static images taken from 3D z-stacks, which are presented fully in Videos 2 and 5, respectively. Insets in B and D show images taken from later time points (identically scaled) to show low photobleaching. All 2D images presented are maximal-intensity projections of a z-series.

**Figure 6.**
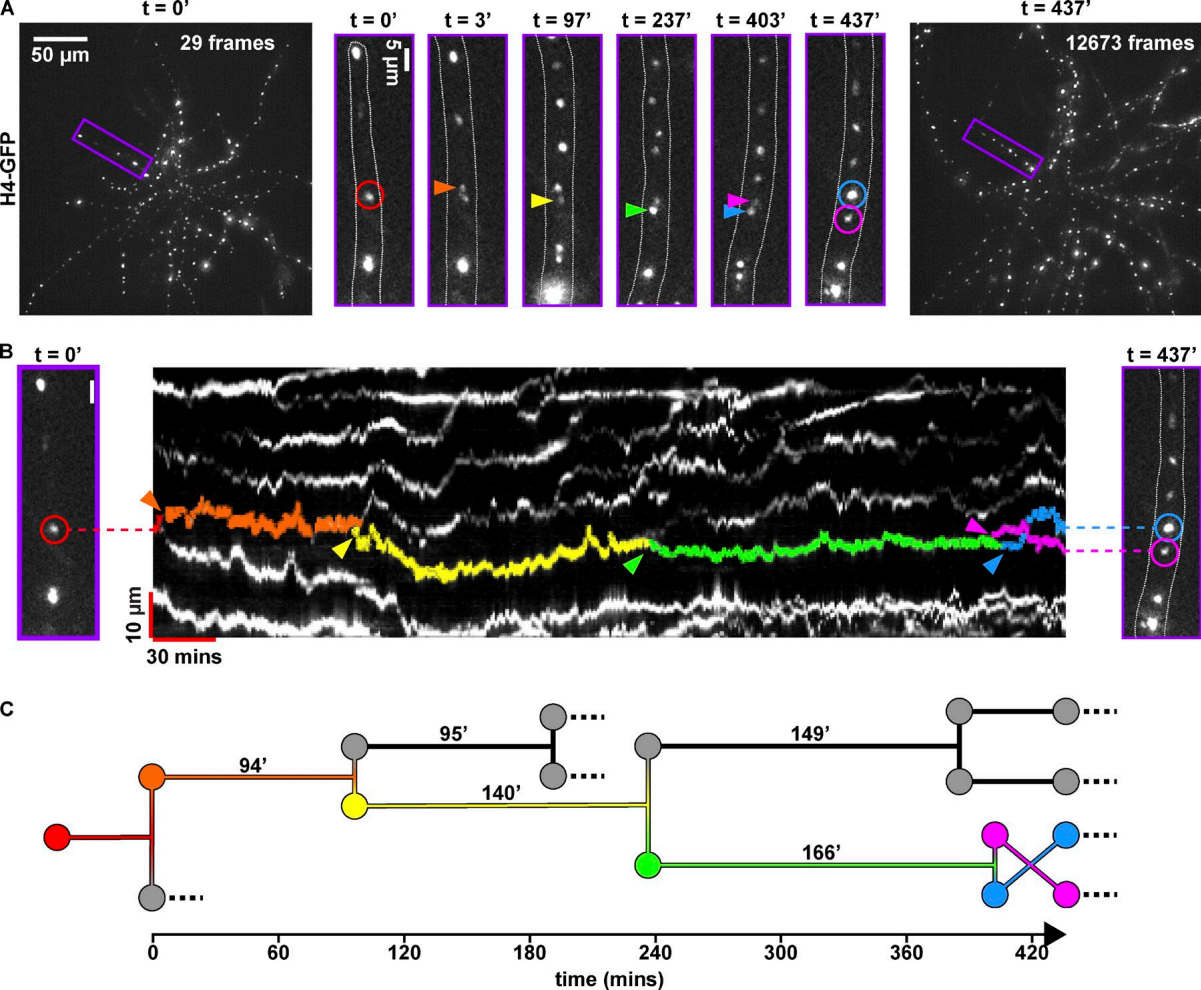
**Long-term nuclear pedigrees. (A)** Pedigrees are shown in *A. gossypii*. Initial (left image), final (right image), and selected subsets (purple-outlined boxes, middle six images) of a 7-h time lapse of *A. gossypii* nuclei. Purple box in left-hand image (t = 0’) is magnified and displayed to the right, outlined in purple. Outlines of hypha are shown as dotted white lines in each subset. Red circle denotes the parent (first-generation) nucleus to be tracked throughout time lapse. Images shown at 3′, 97′, 237′, and 403′ denote times of mitotic division events (anaphase or telophase) of the parent nucleus (3′) or its descendants (97′, 237′, 403′, 437′). Birth events (mitoses) of tracked nuclei in images are denoted with orange, yellow, green, and blue/pink arrows, respectively. The image at 437′ denotes final image of hypha acquired during the timelapse, with blue and pink arrows denoting location of fifth-generation nuclei. All images were taken with a 60× 1.4-NA oil-immersion objective with a 500-ms exposure per frame, 0.5-µm z-steps, and a 14-µm z-range. Images were deconvolved with eight iterations of a Richardson-Lucy deconvolution algorithm. **(B)** Kymograph of the region of interest around hypha from A. Multicolored arrows denote same events as in A. Tracks of nuclei have been false-colored to highlight their lifespans, with the birth of each colored nucleus denoted with colored arrowheads. Note the nuclear bypassing event of blue and pink nuclei at ∼415′. **(C)** Nuclear pedigree tree of the lineage highlighted in A and B. Colored nuclei correspond to the colored arrows shown in A and B, and colored tracks correspond to the false-colored tracks in B. Cell-cycle lengths (as measured by the length of time between mitoses) are indicated in minutes above each nuclear lifespan. Dotted lines indicate nuclei that moved out of the region of interest, could not be tracked or underwent their next division outside of the acquisition timeline.

These data suggest that LITE microscopy imparts less photodamage onto live fluorescent samples while maintaining the resolution and detection efficiency to which cell biologists are traditionally accustomed, a novel combination of benefits that has not yet been achieved by other LSFM modalities. To demonstrate the utility of LITE microscopy with any coverslip-mounted biological sample, we imaged six popular model organisms with various fluorescent markers (Fig. 5, A–E). We selected one plant, *Arabidopsis thaliana* (Fig. 5 C and Video 2); three animals, *C. elegans* (Fig. 5 A and Video 3), *Drosophila melanogaster* (Fig. 5 D and Video 4), and *Hypsibius dujardini* (Fig. 5 E and Video 5); one mammalian cell culture line, HeLa cells (Fig. 5 B and Video 6); and one fungus, *Ashbya gossypii* (Fig. 6 and Video 7) to illustrate the broad phylogenetic spectrum of modern model organisms accessible by LITE. These organisms also exhibit a wide range of sizes, from ∼30 µm (Fig. 5 B, HeLa) to ∼1 cm (Fig. 5 C, *A. thaliana* seedlings) in maximal length. In *C. elegans* expressing a fluorescently tagged kinesin (MCAK-mNG), chromosomes could be resolved between centrosomes (Fig. 5 A and Video 3). In a human cultured cell expressing a fluorescently tagged kinetochore protein, no phototoxic effects (such as cell-cycle arrest) were observed over 28,826 frames (122 min; Fig. 5 B and inset). In plant cells, SAUR63-YFP decorates the cell membrane and fine intracellular structures, which we can visualize in three dimensions with little-to-no out-of-focus autofluorescence (Fig. 5 C and Video 2). In *D. melanogaster*, punctate and junction-associated fluorescently tagged Axin was observed during embryonic germband extension, which occurred at a normal rate with no detectible photobleaching despite exposure for 22,724 consecutive frames (130 min; Fig. 5 D [and inset] and Video 4). LITE is also compatible with imaging of fixed, fluorescently stained samples such as an adult tardigrade, where staining of actin (green) and the outer cuticle (magenta) reveal the intricate network of muscle fibers (Fig. 5 E and Video 5). Collectively, these data reveal that LITE can be used to visualize these organisms at high native resolution with constant illumination (i.e., no laser shuttering) for >2 h without any observable phototoxic effects (Fig. 5, B and D).

### Long-term imaging with LITE enables nuclear lineage analysis

We next set out to demonstrate the power of combining long-term time lapse imaging with low photodamage and high spatiotemporal resolution. The filamentous fungus *A. gossypii* has emerged as a powerful system in which to study syncytial cell biology (Roberts and Gladfelter, 2015). Despite existing in a common cytoplasm, *Ashbya* nuclei proceed through the cell cycle out-of-sync with each other. Previous statistical analyses investigating the source of nuclear asynchrony in *Ashbya* have been based on only single pairs of sister nuclei born of a single mitotic event (Gladfelter et al., 2006; Nair et al., 2010), limiting robust statistical analysis of division patterns across multiple generations. However, long nuclear cycles (between 40 and 200 min; Nair et al., 2010) and highly oscillatory nuclear motions (Anderson et al., 2013) in *Ashbya* necessitate high spatiotemporal resolution, 4D imaging for at least two iterations of the average nuclear cycle (∼3 h) to trace lineages across multiple nuclear generations. To date, tracking nuclei for this duration at high spatiotemporal resolution has been confounded by photobleaching and phototoxicity. To overcome these limitations, we used LITE to image *Ashbya* and track nuclear motion and mitotic asynchrony continuously for >7 h. We expressed a fluorescent histone (H4-EGFP) in *Ashbya* to detect nuclei for measuring motion and division (Fig. 6 A and Video 7). Nuclear divisions in a hypha (Fig. 6 A, purple boxes) were readily identified and tracked for five generations (colored arrows). After 437 min of imaging, the *Ashbya* cell was alive and not detectably photobleached (Fig. 6, A and B; and Video 7). Kymograph analysis enables us to create a temporally scaled pedigree of the nuclear generations (Fig. 6 C). In sum, LITE is a powerful approach for long-term, high spatiotemporal resolution live imaging.

## Discussion

Traditionally, LSFM has been used to reduce photodamage to live fluorescent samples by reducing the illumination to only the focal volume of the detection objective (Santi, 2011), but its geometry has prevented the use of high-NA objective lenses. LITE is the first live-cell SPIM/LSFM modality that allows the use of any objective, allowing researchers to take full advantage of the efficiency of high-NA objectives. If, for example, LITE is used with a 1.49-NA oil-immersion objective, this setup accepts 88% more emitted fluorescence and offers 26% more in native lateral resolution (Fig. 3) than the 1.1-NA water-dipping objective currently used with the lattice light sheet (Chen et al., 2014). Collecting more light affords LITE the ability to generate brighter images, which in turn allows the user to illuminate the sample with proportionally less laser power to collect the same number of emitted photons as with other SPIM/LSFM modalities, which in turn lowers the photobleaching rate (Fig. 4). The high native spatial resolution of LITE (Fig. 3) will allow cell biologists to obtain images with the spatial resolution to which they are accustomed without sacrificing (and, likely improving) temporal resolution, because LITE does not require deconvolution of multiple structured views as does structured illumination microscopy.

Recently, two independent techniques have been presented with tilted illumination modalities similar to LITE. Highly inclined and laminated optical-sheet (HILO) microscopy uses a tilted sheet that is generated from the detection objective (Tokunaga et al., 2008) rather than from a secondary excitation objective. However, HILO is significantly more restricted in its illumination area (15–45 µm) compared with LITE (50–600 µm) because of the significantly higher illumination angle (13°) compared with LITE (1–4°). Additionally, the reported sheet in HILO (6–10 µm) is significantly thicker than the observed thickness of the LITE sheet (4.3 µm; Fig. 2). The second, more recent method, TILT3D, is complementary to LITE in that TILT3D uses a tilted light sheet for the purpose of optical sectioning near the coverslip by using high-NA detection objectives (Gustavsson et al., 2018). Unlike LITE, TILT3D has achieved 3D super-resolution of fluorescent molecules in fixed samples (Gustavsson et al., 2018). Although TILT3D achieves a thinner (2.1 µm) light sheet than our example LITE sheet (4.3 µm; Fig. 2 C), TILT3D does so with two optical disadvantages (in our view): a significantly higher tilt angle (10°) than our LITE sheet (2.4°; Fig. 1 C) and a significantly reduced light-sheet length (73 µm) than our LITE sheet (296 µm; Fig. 2). This reduction in the TILT3D FOV to decrease the light-sheet width is a necessary trade-off, but it significantly reduces the useful FOV of the detection objective (Gustavsson et al., 2018). We elected to demonstrate LITE with a thicker, longer sheet (Fig. 2) for larger samples (Fig. 5, C–E). However, through our implementation of cosine wave optics (Golub et al., 2015), LITE can, in principle, achieve the same 2.1-µm sheet thickness as TILT3D with a sheet of similar length (63.8 µm; Eq. 4) and a significantly lower tilt angle (5.0°; Fig. 1 C).

In addition to high-NA objectives, LITE is also compatible with several other common aspects of modern microscopy. LITE can be installed nonobtrusively on any upright or inverted stand, allowing the use of standard equipment, such as eyepieces, objective turrets, and transillumination (Fig. S3). The compatibility of LITE with microscope stands/stages that differ from the materials used in this paper (Table S2) is dependent on the ability of the stand to stably maintain the detection objective’s position relative to the static, tilted LITE sheet. Additionally, if multiplane acquisition is desired, we strongly recommend a vertical piezo stage with low drift so that repositioning the sample over long periods of time is possible. Because the native point-spread function of LITE is identical to that of epi-illumination (Fig. 3), standard postacquisition deconvolution algorithms can be used on LITE images just as with epi-illumination. For example, the Richardson-Lucy deconvolution algorithm was used for our *Ashbya* images to increase the contrast between the nuclei and the cytoplasm. Any standard epi-illumination deconvolution algorithm that is based on a detection objective’s point spread function is suitable to increase contrast in LITE images.

LITE produces less photobleaching than epi-illumination, both in the rate at which fluorophores photobleach (Fig. 4 C) and the preservation of the image contrast over the acquisition time (Fig. 4 B). By selectively illuminating a thin slice of the sample (Fig. 2), LITE reduces the background (a combination of out-of-focus signal and out-of-focus autofluorescence) relative to the in-focus signal, thus increasing the overall image SBR. High SBR provides high contrast of the structure of interest from the confounding out-of-focus background fluorescence, as well as from sample autofluorescence. The higher variability in the LITE photobleaching rates (Fig. 4 D) could be attributed to variability in sheet alignment, chamber construction (Fig. S2 A), or biological noise. Although the LITE sheet measured 4.3 µm thick FWHM (Fig. 2 D), in actual cellular imaging conditions, it behaved as if it were thinner. Because of the complexities of cells, this phenomenon is difficult to measure and is best illustrated by the observation that focusing the detection objective (without moving the sheet) by ∼1 µm resulted in being outside the excitation volume. Although we have no experimental evidence for this observation, it is conceivable that, given the sheet has a Gaussian intensity profile, only the very peak of the focal volume contains a photon density adequate for fluorophore excitation. Regardless of the variability in sheet alignment or its functional thickness in living samples, our work demonstrates that LITE can be used to image fluorescent samples for longer periods of time than with epi-illumination (Fig. 4 D and Video 1).

As has been observed with current LSFM designs (Huisken et al., 2004; Santi, 2011; Chen et al., 2014; Wu et al., 2016), we found that LITE decreases the fluorophore bleaching rate compared with epi-illumination (Fig. 4, Fig. S4, and Video 1). Theoretically, this decrease could allow users to reach an equilibrium between photobleaching and turnover at a higher signal and higher SBR with LITE than with epi-illumination. Furthermore, we observed an intriguing phenomenon in several of our model organisms in which fluorescence intensity does not detectably decrease over the course of the time lapse (Figs. 5 B, 5 D, and 6, corresponding to Videos 6, 4, and 7, respectively). To explain this phenomenon, we suggest that addition of new fluorophores in live organisms could compensate for loss via photobleaching. If the translation, maturation, and loading of unbleached biological fluorophores collectively result in a simple linear increase in fluorescence, fluorophore turnover could compensate for most photobleaching in live-cell fluorescence microscopy, provided the photobleaching rate is low enough. Understanding this phenomenon will require further study, as it requires characterization of protein abundance and turnover rates to accurately calculate the photobleaching rate in living, developing samples.

We are confident that the decreased rate of photobleaching that LITE offers will allow cell biologists to observe intracellular dynamics at higher native spatiotemporal resolution and for significantly longer periods of time than previously possible using other modes of fluorescence microscopy. We have demonstrated one application of LITE in tracing nuclear lineages (Fig. 6). Lineage tracing has powerful implications, because asymmetric and symmetric inheritance of factors that determine cellular behavior is integral in determining how cells born of a single ancestor can differentiate to different fates.

In the past, we have used the model fungal system *A. gossypii* where nuclei divide asynchronously in a common cytoplasm (Gladfelter et al., 2006). Previous work has found that individual nuclear cycles in a single *Ashbya* cell can vary significantly in their timing (Gladfelter et al., 2006), suggesting that there exists nuclear-intrinsic and/or -extrinsic factors that influence nuclear timing. A limitation of past nuclear tracking experiments (Nair et al., 2010; Anderson et al., 2013) was that photobleaching and phototoxicity prevented long-term imaging that would allow collection of nuclear lineage data over multiple generations, limiting the ability to robustly test for lineage-dependent similarities in nuclear timing.

With LITE microscopy, we are now able to image nuclei for >7 h to visualize multiple rounds of nuclear division with no noticeable photodamaging effects (Fig. 6 and Video 7). These data will allow us to study the heritability of division timing over several generations and further our understanding of how heritable nuclear-intrinsic signals contribute to division asynchrony in *Ashbya*. These sorts of extended image series and statistical analyses are relevant to establishing lineages and division patterns in any cell type, from stem cells to tissues.

Beyond tracking nuclei in *Ashbya*, we demonstrate that LITE can be effectively used to visualize fluorescent labels in a wide variety of organisms at high native spatial resolution. With LITE, cell biologists can now image without photodamage far longer than with conventional modes of fluorescence microscopy. In addition, cell biologists can reduce the photodamage to their samples without sacrificing spatial resolution or detection efficiency. Thus, LITE allows biologists to observe practically any live, fluorescent organism with unprecedented efficiency and resolution for previously unattainable periods of time. These newfound observations with the use of LITE will undoubtedly allow biologists to better understand the intricacies of cellular and subcellular dynamics.

## Materials and methods

LITE is a novel method for introducing a light sheet within the working distance of high-NA objective lenses for live-cell fluorescence microscopy (Fig. 1 A). In brief, these goals were accomplished by first directing a collimated, coherent beam of excitation light through a photomask and cylindrical lens. The cylindrical lens focused the excitation light to form a roughly “wedge-shaped” beam of light. The beam converged to its minimal thickness and formed the light sheet at the focal plane of the cylindrical lens, ∼3 cm away from the cylindrical lens. The photomask was used to pattern the focusing beam so that the light sheet was lengthened (Golub et al., 2015). To access the working distance of high-NA lenses, the excitation light was tilted such that the bottom of the converging wedge was parallel to the detection objective focal plane. Thus, the light sheet was formed at the focal plane of the detection objective, in which the fluorescent sample was mounted. 3D imaging with LITE was made possible by supporting sample chambers on a vertical piezoelectric motorized stage and moving the sample through the sheet. LITE allows mounting samples on coverslips, provided the chambers also have an optically clear opening to allow access by the converging illumination light. We have engineered several suitable chambers and present imaging data from a diverse range of model organisms. We have included the parts list (commercial and custom parts) and assembly instructions for the LITE system in the supplemental materials (Tables S1 and S2 and Data S1 and S2).

### Illumination

LITE imaging requires collimated, radially symmetric, coherent illumination light. We generated such a beam using a collimator illuminated by a laser combiner (Monolithic Laser Combiner 400; Agilent Technologies) with a fiber connector/angled physical contact fiber-coupled laser output of four wavelengths (405, 488, 561, and 650 nm). The four laser sources were solid state and prealigned to deliver a radially symmetric, coherent beam (Fig. S1). The maximal power outputs, after the fiber, of the four lasers in order of increasing wavelength were 18, 52, 55, and 37 mW, although only a fraction of each beam is used to generate the light sheet. The choice of illuminator should be based on specific application, fluorescent proteins in vivo in this case. An internal acousto-optical-tunable filter, analogue-controllable via DAQ Board interface, was used for modulating wavelength intensities. For brevity, we mainly describe our setup as monochromatic illumination at 488-nm excitation (for EGFP), although we outline two alternative methods for multicolor LITE imaging in Data S3.

### Beam conditioning

LITE illumination involves conditioning from the laser source such that the diameter of the radially symmetric beam is magnified to a value that is equal to or greater than the full aperture of the slits of a customized photomask (see the next section). The beam should remain collimated after conditioning. Here, collimation and beam expansion were combined by a fiber connector/angled physical contact–coupled total internal reflection fluorescence microscopy collimator (Nikon Instruments) that achromatically collimated the lasers to a beam diameter of 22 mm (Fig. S1).

### Photomask/cylindrical lens system

We used a cylindrical lens to focus a radially symmetric, collimated beam along one axis to approximate a nondiffracting “sheet” of light at the focus of the cylindrical lens. The sheet itself (in the focus of the cylindrical lens) can be approximately defined as a rectangular prism with three dimensions: the thinnest, diffraction-limited vertical width (*w*) that the converging laser reached at the cylindrical lens focal plane, the axial length (*L*) over which the laser remained at its diffraction-limited width before diverging, and the unfocused horizontal breadth (*b*) of the laser. The FWHM of the sheet (hereafter referred to as *w*) is defined by Eq. 1:

$$w=\frac{n\lambda_{ex}\sqrt{2\ln2}}{\pi NA_{eff}\;},$$ (1)

where *n* is the refractive index of the medium in which the laser was focused to a sheet (typically ∼1.33 for aqueously media, although this value varies based on the temperature and chemical composition of the media and the wavelength of the excitation light), *λ_ex_* is the wavelength of the excitation laser (in micrometers), and *NA_eff_* is the effective NA of the cylindrical lens. Note that *NA_eff_* can be smaller than the reported NA of the cylindrical lens, because *NA_eff_* depends on the percentage of the cylindrical lens NA that is used (i.e., the vertical height of the collimated excitation light incident on the cylindrical lens back aperture). Thus, *w* is inversely proportional to the diameter of the collimated beam incident to the cylindrical lens, assuming the beam diameter is less than the full cylindrical lens back aperture. The thinnest sheet possible is preferable in traditional LSFM, for two reasons: (1) to minimize out-of-focus excitation/emission in the fluorescent sample and (2) to prevent photodamage in out-of-focus planes. However, the choice of sheet thickness in LITE was complicated by the mathematical interdependence of *w* and *L*, in Eq. 2:

$$L=\frac{\pi w^{2}}{2\lambda_{ex}\;}.$$ (2)

As shown in Eq. 2, it is evident that *L* increases with the square of *w*. Practically, this meant that the thinnest sheet possible (minimal *w*) was not necessarily the best sheet for LITE, because the distance over which the sheet remains diffraction-limited (*L*) could have been too short to cover the *FOV* of the detection objective used for detecting the signal. If the sheet began to diverge over the *FOV*, then the illuminated slice of the fluorescent sample would vary significantly in both thickness and illumination intensity along the *FOV*. This would result in inconsistent excitation of fluorophores, making quantitative analysis of fluorescent images difficult.

To maximize the *L* for a given *w*, we placed a quadruple-slit photomask (FrontRange Photomask) in the principle plane of the cylindrical lens, before the beam enters the lens (Fig. S1). The theoretical and practical design of these slits were first described and implemented by Golub et al. (2015). In brief, this method increased *L* of a cylindrical lens-based light sheet beyond what Eq. 2 predicts by creating an interference pattern at the cylindrical lens focal plane between two harmonic cosine waves (Golub et al., 2015)_._ Golub et al. (2015) presented the equation for the depth of field (DOF) of the elongated light sheet in Eq. 3:

$$L'=\frac{\lambda_{ex}f^{2}}{R_{1}{}^{2}},$$ (3)

where *L′* is the elongated sheet length, *f* is the cylindrical lens focal length, and *R_1_* is the radius of the inner photomask slits (Golub et al., 2015). To put Eq. 3 in terms of *w*, we equated *R_1_* to *NA_eff_* using Eq. 1 and substituted the equivalence into the Eq. 3 denominator to arrive at Eq. 4:

$$L'=\frac{\lambda_{ex}}{\tan^{2}\left[\sin^{-1}\left(\frac{\lambda_{ex}\sqrt{2\ln2}}{\pi w}\right)\right]}.$$ (4)

In LITE as described here, the thickness and spacing of the photomask slits were scaled from the values for a 152-mm-focal-length cylindrical lens (Golub et al., 2015) to the scale of our selected 40-mm-focal-length, aspheric, cylindrical lens (AYL5040-A; ThorLab). The optical trade-off of this interference strategy was the generation of side lobes and loss of illumination intensity. Side lobes should theoretically manifest as coplanar light sheets above and below the bright center peak of the main light sheet. However, >80% of the total laser energy should remain in the center sheet, because the side lobes destructively interfere (Golub et al., 2015). Side-lobe minimization is important to reduce the probability of excitation and emission outside the detection objective focal plane.

### Optimization of sheet dimensions and parameters

Creating a nondiffracting light sheet of a width within an order of magnitude of the wavelength of light requires that the light be focused. Accordingly, previous light-sheet fluorescence microscopes have used standard (or custom) objective lenses to focus a beam to create a light sheet of a minimal width in the sample (Huisken et al., 2004; Santi, 2011; Chen et al., 2014; Wu et al., 2016). This orthogonal, two-objective method sterically limits the choice of detection objectives to those with a long-enough working distance (>1 mm) to focus on the sheet, because the illumination and imaging objectives cannot touch. Here, we present a novel solution for using virtually any existing microscope objective, including those with high NA, for imaging fluorescence signal from a light sheet (Fig. 1 A). This represents a significant advance in LSFM, because biologists are no longer limited in their choice of objectives (Fig. 1 B). A detailed, a step-by-step method for selecting the ideal setup of a LITE microscope illuminator based on the desired objective is presented below.

For effective imaging with LITE, it is necessary to illuminate an objective’s volume of view (VOV) while minimizing illumination outside the VOV. An objective’s VOV can be defined by the product of the 2D *FOV* and the 1D *DOF*. The *DOF* of an objective, otherwise known as axial resolution, is a set parameter that varies based on the NA and wavelength of the emitted fluorescence (*λ_em_*) that is collected by the objective (Eq. 6).

The relationship between the light-sheet FWHM thickness, *w*, and the detection objective *DOF* was derived from the necessity to form the light sheet at the coverglass surface so that it is within the working distance of high-NA objectives. Confined by this geometry, it is impossible to form a light sheet that is completely orthogonal to the focal plane of a high-NA objective within its standard working distance (typically <300 µm) while also projecting the converging beam over a flat surface, such as a coverslip. To solve this problem, we tilted the collimated beam, photomask, and cylindrical lens relative to the surface of the objective. Tilting in LITE was done at a precise, but customizable, angle: the half angle of the laser as it converges in aqueous media. Tilting the LITE setup at this half angle, *θ*, allowed the bottom part of the converging beam to propagate parallel to the coverslip surface without reflection or refraction of the laser through the coverslip before the laser reached the sample (Fig. 1 A). Tilting a light sheet relative to the objective’s *FOV* is not typical of traditional live-cell light sheet modalities (Huisken et al., 2004; Santi, 2011; Chen et al., 2014; Wu et al., 2016). Because of this aspect of our design, it is distinct from current live-cell LSFM methods.

To determine the ideal light-sheet dimensions (*w*, *L*, and *θ*) from the parameters of any desired objective (magnification *M*, *NA*, *FOV*, and *DOF*), several basic mathematical relationships were considered. We first determined a useful relationship between *w* and an objective’s *FOV* and *DOF*. To calculate the full *FOV* of the desired objective, Eq. 5 was considered:

$$FOV=\frac{FN}{M},$$ (5)

where *FN* is the field number of the objective (in micrometers) and *M* is the lateral magnification of the objective (dimensionless). Thus, *FOV* is the full 1D diameter (in micrometers). If a shorter *FOV* were desired (e.g., the length of a camera’s pixel array), it could be instead defined manually as some fraction of the full objective *FOV*. Next, to calculate the theoretical *DOF* of a desired objective, Eq. 6 was considered:

$$DOF=\frac{1.61n_{im}\lambda_{em}}{NA^{2}}.$$ (6)

The variables in Eq. 6 (*n_im_* and *λ_em_*) correspond respectively to the coverslip-immersion medium refractive index and fluorophore peak emission wavelength (in micrometers). It is worth noting that the useful *DOF* of an objective (axial resolution) changes based on the desired fluorophore, because both *λ_em_* and *n_im_* vary based on the fluorophore.

Once the *DOF* and *FOV* have been correctly identified for the objective of choice, the ideal width of the light sheet was calculated. Eq. 7 was derived to determine the dependency of *w* on *DOF*, *FOV*, and *λ_ex_*:

$$w=\sqrt{\left\{\begin{array}{c} \frac{DOF^{2}}{2}+\frac{\sqrt{2\ln\left[2\right]}\lambda_{ex}FOV^{2}\sqrt{\frac{DOF^{2}+FOV^{2}}{FOV^{2}}}}{\pi \sqrt{DOF^{2}+FOV^{2}}}+\ldots \\ \ldots \frac{1}{2}\sqrt{\left\{DOF^{4}-\frac{8\ln\left[2\right]{\lambda_{ex}}^{2}DOF^{2}}{\pi^{2}}+\frac{4\sqrt{2\ln\left[2\right]}\lambda_{ex}DOF^{2}FOV^{2}\sqrt{\frac{DOF^{2}+FOV^{2}}{FOV^{2}}}}{\pi \sqrt{DOF^{2}+FOV^{2}}}\right\}} \end{array}\right\}}.$$ (7)

Thus, we arrived at a function of two variables such that *w* = *f* (*FOV, DOF*). In sum, the ideal light-sheet width for any given objective could be calculated. To illustrate the general trend of how *w* varied as a function of objective parameters, we obtained the *FOV*, *M*, *NA*, and *DOF* of 90 commercially available detection objectives and plotted the optimal light-sheet thickness (*w*) for each objective as a function of its *NA* (Fig. 1 B).

Once the width of the light sheet was known, we then calculated the length *L′* over which a light sheet of that width remains nondiffracting from Eq. 4. Finally, we also calculated the half-angle of the converging laser, *θ*, that forms the light sheet, by substituting the general equation for lens NA (*NA* = *n*sin(*θ*)) into Eq. 1 and solving for *θ* to yield Eq. 8:

$$\theta =\sin^{-1}\left[\frac{\sqrt{2\ln2}\lambda_{ex}}{\pi w}\right].$$ (8)

The resultant angle from Eq. 8 is the maximal angle at which the focused sheet should be tilted relative to the VOV inside the sample chamber. Our selected cylindrical illuminating lens was a dry lens that focuses the laser into air (*n* ≈ 1), so the laser must first refract into the sample chamber (*n* ≥ 1.33) before reaching the sample (see Sample chambers). Eq. 8 is plotted in Fig. 1 C to visually illustrate that *θ* decreases exponentially as *w* increases. Because *θ* and *w* vary with respect to the different excitation and emission wavelengths among fluorescent proteins, five traces are shown in Fig. 1 C that correspond to five commonly used biological fluorophores (BFP, CFP, GFP, YFP, and mCherry) and their respective maximal excitation wavelengths (383, 433, 488, 513, and 587 nm). All equations are presented in Table S1 for automatically calculating all relevant LITE parameters for a desired detection objective.

If the tilting is kept to the minimum *θ* necessary to completely illuminate the *FOV* of interest, then out-of-focus excitation was still dramatically reduced (compared with conventional illumination) in the case of all objectives over a wide range of NAs, magnifications, and DOFs (Fig. 1, B and C). A byproduct of this scheme was that ***w*** was always wider than the DOF, a feature that (in principle) lead to increased out-of-focus excitation compared with conventional light-sheet illumination. However, in part because of the optical sectioning ability of high-NA lenses and the Gaussian nature of light-sheet intensity, this effect was not observed in practice (see Discussion). In addition, the light-sheet length and width have a wavelength dependence, which should be considered for multichannel imaging. Practically, one preferred excitation wavelength can be selected, thus keeping the photomask and tilt angle constant while still allowing similarly shaped light sheets of different wavelengths. Multicolor LITE imaging is discussed further in Data S3.

### Sample chambers

To be compatible with LITE, sample chambers must meet two main criteria: (1) have a glass coverslip as the bottom surface for use with high-NA objectives and (2) have a flat, optically clear, and homogenous side to allow the laser to focus inside of the chamber at the coverslip surface. Images presented in this paper were acquired by using one of two types of chambers that meet these criteria.

The first type of chamber (Fig. S2 A, hereafter referred to as chamber A) consisted of an open-topped, media-filled box formed by using four 22 × 22 mm #1.5 crown glass coverslips and one 24 × 60 mm #1.5 crown glass coverslip. The coverslips were cemented in place by using VALAP, a 1:1:1 mixture wt/wt of Vaseline, lanolin, and paraffin. For the laser to enter the chamber normal to the front coverslip surface, it was necessary to angle the front coverslip at the sheet convergence angle, *θ*. Advantages of chamber A included short fabrication time (∼3 min) and low cost per unit. Disadvantages of chamber A included incompatibility with samples less dense than their media (samples do not sit on the surface of the coverslip while immersed in media), incompatibility with upright microscopes, irreproducibility of the tilt angle of the front coverslip, irreproducibility of sample positioning, and potential VALAP leaking into the chamber that interferes with the converging laser.

To overcome some of the disadvantages of chamber A, a second chamber was created (Fig. S2 B, hereby referred to as chamber B). Chamber B was a microfluidic chamber consisting of a 24 × 60 mm #1.5 glass coverslip and an imprinted piece of polydimethylsiloxane (PDMS; commercially available as Sylgard 184 from Corning; see Fig. S2 C for a 2D view of the imprint pattern). Two copies of the imprint pattern were copied onto a photomask for photolithography (FrontRange Photomask). Templates for microfabrication were made by spin-coating 1002-F negative photoresist (Pai et al., 2007) onto clean 50 × 75-mm glass slides at various speeds (1,500–3,000 rpm) for various thicknesses of photoresist (7–50 µm). Slides were exposed to 400–600 mJ UV radiation under the patterned photomask. Unpolymerized photoresist was removed chemically, leaving behind hardened microfeatures on the template that act as a negative for imprinting PDMS. The template was placed inside a sealed metal casting chamber with two polished, flat metal sides, tilted at *θ* relative to the normal of the template’s surface. Premixed liquid PDMS (1:10 wt/wt ratio of cross-linker to base) was cast over the template before vacuum degassing. PDMS was heated to 40°C and left to polymerize for 24 h before separation from the template. We used a 1.0-mm biopsy punch to create inlet and outlet channels through the PDMS into the microchamber for flowing in media/samples. PDMS molds were cleaned with methanol, ethanol, and distilled water, and coverslips were cleaned with isopropyl alcohol and acetone before both the molds and the coverslips were plasma treated for 30 s. PDMS molds were bonded by physical adhesion to coverslips to create the finished chamber B. Advantages of chamber B included high reproducibility among chambers (in shape, size, and tilt angle *θ* of the side), high customizability, the ability to mount any sample in a reproducible location (close to the coverslip, in the focused sheet), and compatibility with samples of low density (e.g., *A. gossypii*). The main disadvantage of chamber B was a longer production time per unit (24 h).

### Microscope hardware parameters

LITE microscopy can be used on a broad diversity of microscope stands (inverted or upright), with any objective, and with any coherent, collimated laser source. The physical setup of our LITE prototype is detailed below, but it may be easily adapted for different existing microscope hardware. The LITE apparatus was constructed adjacent to a TE2000 inverted stand (Nikon Instruments; Fig. S3). The stand is equipped with an XY motorized stage (50-mm travel; Prior Instruments) for positioning of samples and a piezo-motorized Z stage (100-µm travel; Prior Instruments) for scanning the sample through the light sheet/focal plane during multiplane acquisition.

Several custom parts necessary to position the photomask, cylindrical lens, and collimator at the appropriate angle relative to the detection objective were designed by using AutoCAD for Mac 2015 (AutoDesk) and were either manufactured by using a 3D printer or machined from aluminum. All 3D printing was performed with a uPrint SE (Stratasys) using acrylonitrile butadiene styrene plastic (courtesy of Kenan Science Library Makerspace, University of North Carolina at Chapel Hill [UNC-CH]). All custom machining was performed by the Physics and Astronomy Instrument Shop (UNC-CH). Computer-assisted design (CAD) files of custom parts are available in Data S1. Assembly instructions are available in Table S2 and Data S2.

For fluorescence detection/magnification, a variety of detection objectives was used. All objectives used in this article for fluorescent organism visualization are coverslip-based, water or oil immersion, NA ≥1.2, infinity corrected, with magnifications between 40 and 100×. Specific objective parameters for individual image sets are listed in the figure legends. A 535/50-nm emission filter was installed in the infinity path of the objective to filter out scattered 488-nm excitation light (Fig. S1). No other filters (e.g., dichromatic mirrors) are necessary in LITE. Magnified images were refocused with a 1× tube lens onto an Andor Zyla 4.2 sCMOS camera. Laser acousto-optical tunable filter (described in Illumination in Materials and methods), motorized Z piezo position, and camera firing were trigged through a DAQ board interface (National Instruments) and controlled through NIS-Elements (Nikon Instruments).

### Sample preparation

All *C. elegans* specimens were cultured on nematode growth media plus 2% agar petri dishes and fed with OD421 bacterial cultures over 3 d at 20°C. Adult *C. elegans* were dissected in M9 media (17 mM K_2_HPO_4_, 42 mM Na_2_HPO_4_, 85 mM NaCl, and 1 mM MgSO_4_) to obtain embryos, which were mounted in chamber A with M9 or M9 plus 2 mM NaN_3_ (for photobleaching measurements). Strains used include LP148 (*unc-119(ed3) his-72(cp10[his-72::gfp+ LoxP unc-119(+) LoxP])* III; Dickinson et al., 2013) and LP447 ((cp178[klp-7::mNG-C1^3xFlag]) III; Heppert et al., 2018).

HeLa cells stably expressing Hec1-EGFP were provided courtesy of E. Salmon (UNC-CH, Chapel Hill, NC). HeLa cells were cultured in DMEM (Thermo Fisher Scientific) supplemented with 10% FBS (Sigma), 100 U/ml penicillin, and 100 mg/ml streptomycin at 37°C in a humidified atmosphere with 5% CO_2_. Rounded mitotic cells were shaken off 1 h before imaging and mounted in L-15 medium (Thermo Fisher Scientific) in a poly-l-lysine–coated (Sigma) chamber A. Cells were kept at ∼32°C while imaging by using a heated fan. Heating equipment was provided courtesy of E. Salmon.

*A. thaliana* samples were provided courtesy of J. Reed and P. Nagpal (UNC-CH, Chapel Hill, NC). Seeds were surface sterilized and plated onto 0.5× Murashige and Skoog salts (Murashige and Skoog, 1962) and 0.6% Phyto Agar (Research Products International) plates. Germination was induced by incubation at 4°C for 48 h when plates were moved to a growth incubator equipped with a mix of fluorescent and incandescent lights and set to 23°C. After 5 d, seedlings were mounted in chamber A coated with poly-l-lysine (Ted Pella, Inc) and covered with a thin slab of 2% agarose before the chamber was filled with distilled water.

*D. melanogaster* adults were mated for 3 d at 25°C on apple juice plates. Embryos were collected 3–5 h after egg laying and dechorinated in 50% bleach for 5 min. Embryos were then sorted under a dissecting scope, by looking for embryos at pre- or early germband extension. Embryos were then mounted on a poly-l-lysine (Ted Pella, Inc)–coated coverslip and coated with Halocarbon oil 700 (Lab Scientific) in chamber A (Fig. S2 A) for imaging. The following stocks were obtained from the Bloomington Stock Center: *Maternal α tubulin GAL4* (7062) and UAS-Axin:GFP (7225).

*H. dujardini* tardigrade cultures were maintained in 2-liter flasks with oxygenation by using spring water (Poland Spring) as culture media and fed *Chlorococcum* sp. algae. To isolate specimens, a small amount of culture was decanted into a 60-mm petri dish, and animals were transferred to a 1.5-ml microcentrifuge tube by using a dissecting microscope and mouth pipet. Specimens were relaxed in carbonated water for 1 h before fixation. Specimens were fixed in 4% EM Grade Paraformaldehyde (Electron Microscopy Sciences) in PB-Triton (1× PBS and 0.1% Triton X-100, pH 7.4) for 15 min at room temperature (Smith and Jockusch, 2014). Specimens were used immediately for staining. Fixed specimens were stained with phalloidin and WGA. WGA labels the cuticle of *H. dujardini*. Alexa Fluor 594 WGA (Thermo Fisher) was diluted to 10 µl/ml in PBS, and specimens were incubated in this solution overnight. After WGA labeling, specimens were washed four times for 15 min and then left overnight in PB-Triton with 0.1% NaN_3_. Specimens were incubated for 1 d in a 1:40 dilution of phalloidin (Oregon Green 488 conjugated; Molecular Probes) in PB-Triton with 0.1% NaN_3_ and then washed three times for 5 min in PB-Triton (Smith and Jockusch, 2014). Fixed and stained tardigrade adults were kept at 4°C until imaging. Tardigrades were moved to a PBS-filled chamber A and positioned by using mouth pipette for imaging.

*A. gossypii* cells were germinated in chamber B at 30°C for 8 h in *A. gossypii* 2× low-fluorescence media (Yeast base + nitrogen, folic acid, riboflavin [1.7 mg/ml; Sunrise Scientific], 1.6 mg/ml Complete Supplement Mixture [CSM]-ade, 1.0 mg/ml *myo*-inositol, 20 mg/ml dextrose, 7.0 mg/ml aspartic acid potassium salt, 7.0 mg/ml glutamic acid potassium salt, and 10 µg/ml adenine hemisulfate, pH 7.0, with 100 µg/ml ampicillin and 200 µg/ml G418 [Geneticin]). After 8 h, fresh media were added, and chambers were moved to room temperature for imaging.

### Image processing

Images were acquired by using NIS-Elements. Unless otherwise specified, all images presented in this paper are raw acquisition data (after camera offset subtraction). No postacquisition deconvolution or stitching is required to view LITE images, although for some multiplane videos maximal-intensity projections were generated in the z dimension or deconvolved (specified in the figure legends). Fluorescence-intensity measurements, kymographs, maximal-intensity projections, image scaling, false-coloring, and video annotations were performed using Fiji. Richardson-Lucy deconvolution images and 3D videos were made by using NIS-Elements.

### Online supplemental material

The figures include a diagram of the LITE microscope light path (Fig. S1), drawings of sample chambers for LITE (Fig. S2), photographs of the LITE prototype (Fig. S3), and supplemental bleaching comparisons between LITE and epi-illumination (Fig. S4 and Video 1). Supplemental videos include time-lapse and 3D views of fluorescent images presented in Figs. 4, 5, and 6 (Videos 2–7). All relevant equations for building a LITE system are assembled in Table S1. Part descriptions and assembly instructions are presented in Table S2. All custom and commercial part drawings are included in CAD format in the Data S1 file. Visual LITE assembly instructions are presented in the Data S2 file. Multicolor LITE methods are outlined in the Data S3 file.

## Supplementary Material

Supplemental Materials (PDF)

Tables S1 and S2 (ZIP)

Data S1-S3 (ZIP)

Video 1

Video 2

Video 3

Video 4

Video 5

Video 6

Video 7
