# Supplementary material for: LITE microscopy: Tilted light-sheet excitation of model organisms offers high resolution and low photobleaching
Source: J Cell Biol. 2018 May 7;217(5):1869–82. doi: 10.1083/jcb.201710087 (PMC5940309; doi:10.1083/jcb.201710087)
Supplement: Supplemental Materials (PDF) [file JCB_201710087_sm.pdf]

Supplemental material

Fadero et al., https://doi.org/10.1083/jcb.201710087

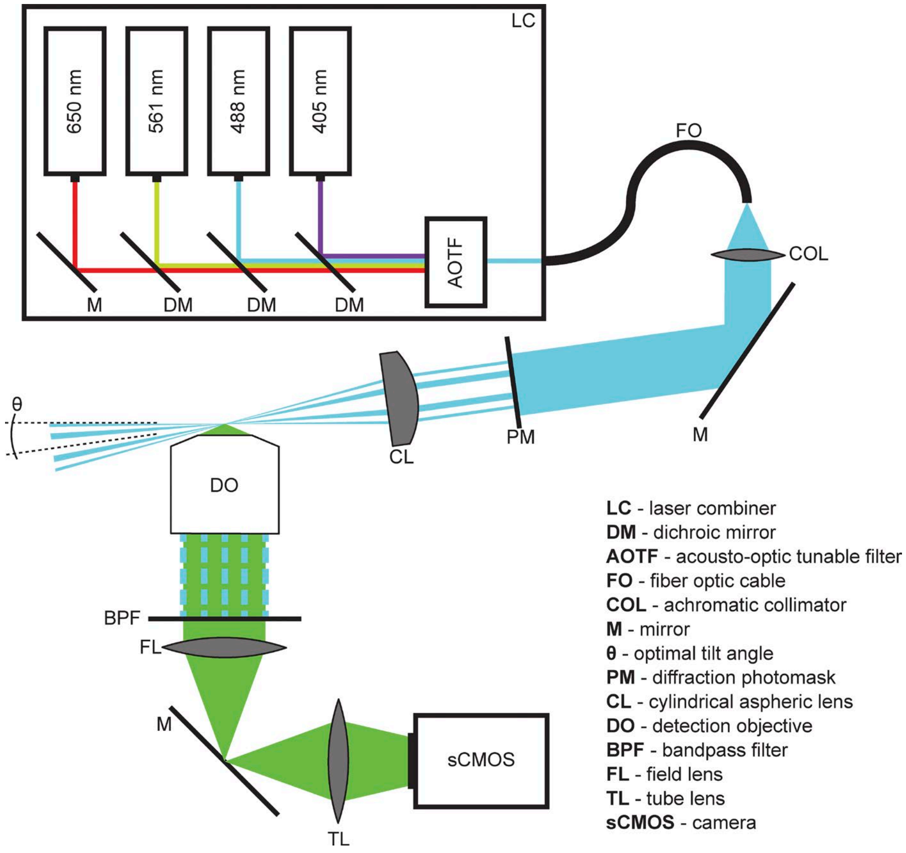

Figure S1. **Schematic of optical path of our LITE microscope.** Objects are not drawn to scale. Our setup has the possibility to be tetrachromatic, but we show this figure as monochromatic with 488-nm (cyan) excitation light and 509-nm (green) emission light to represent a typical GFP acquisition schematic.

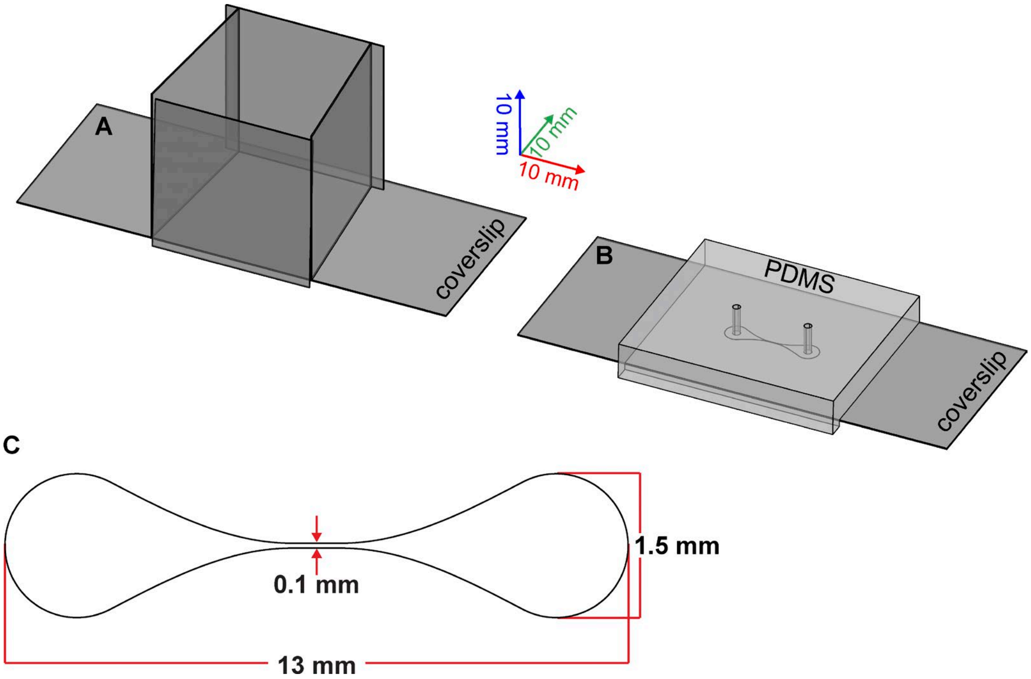

Figure S2. **Sample mounting chambers for use with LITE. (A)** Sample chamber A, constructed of four #1.5 22 × 22-mm coverslips and one #1.5 24 × 60-mm coverslips. The front coverslip is tilted at θ. **(B)** Sample chamber B, constructed of a micro-imprinted PDMS substrate (light gray) and a #1.5 24 × 60-mm glass coverslip (dark gray). The front surface of the PDMS is tilted at θ. In A and B, a 3D scale bar is denoted as three orthogonal vectors along the x, y, and z axes (red, green, and blue, respectively) with 10-mm lengths. The light sheet propagates in the +y direction, and the detection axis is in the −z direction for our inverted setup. **(C)** Enlarged, 2D view of the micropatterned flow chamber in B.

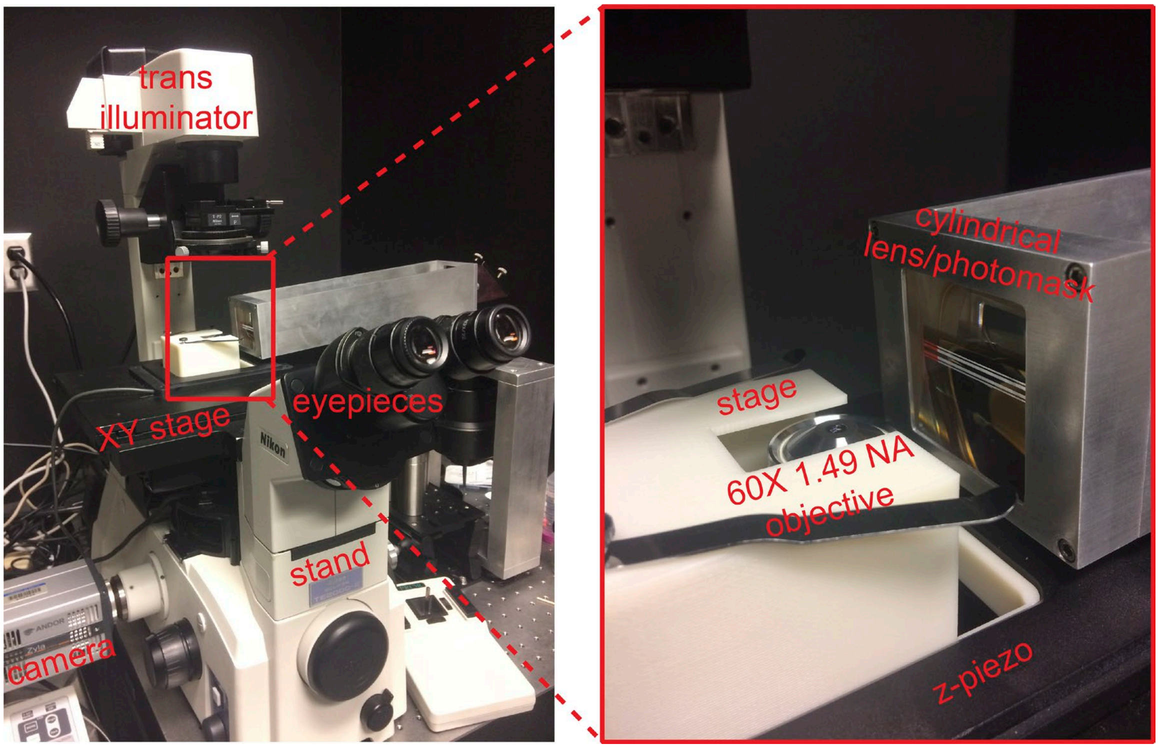

Figure S3. **Annotated photograph of the LITE prototype.** All relevant components are labeled in red, and a zoomed-in view of the stage is outlined in red for a more detailed view of the relative position of the objective relative to the cylindrical lens. Objectives are readily exchangeable.

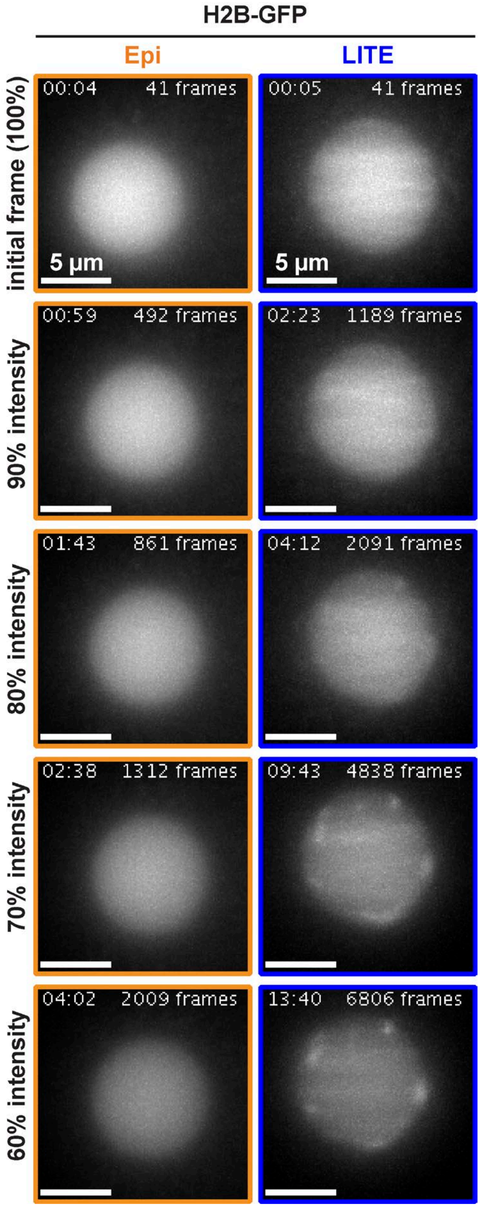

Figure S4. **Image series taken from Video 1.** Images of *C. elegans* (LP148) P1 nuclei shown are selected to illustrate the number of frames acquired (upper right-hand corner of each image) until the nuclei bleached to the specified percentage of the original intensity (left-hand side of each row of images).

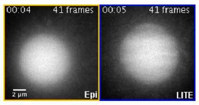

Video 1. **Dynamic comparison of bleaching rates between epi-illumination and LITE in NaN_3-treated *C. elegans* (LP148) embryonic nuclei with fluorescently marked histone H4-EGFP.** Bar, 2 µm. Each nuclear video is internally scaled to the first frame of the video (Fig. 4 A, top images). Total amount of time (top left of each video in minutes:seconds) and the cumulative number of frames acquired (top right of each video) are marked. Each nucleus is identically temporally scaled, because both videos were acquired by using the same acquisition parameters.

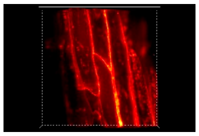

Video 2. **3D projection of an *A. thaliana* seedling expressing a *SAUR63*:YFP construct.** Images were acquired by using a 60× 1.2-NA water-immersion objective with the seedlings mounted in chamber A. Each frame was acquired with a 300-ms laser exposure, a z-step of 1 µm, and a total z-range of 27 µm. Shown is a 3D projection of the stack with a "Red Hot" false coloration (Fiji). The bounding box (dashed white lines) represents 221.87 × 221.87 × 27.0 µm.

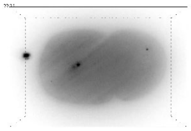

Video 3. **3D projection of multistep time lapse image of 2-cell *C. elegans* embryo (LP447) expressing a recombinant klp-7-mNeonGreen construct.** Images were acquired by using a 60× 1.2-NA water-immersion objective with the embryo mounted in chamber A. Each frame was acquired with a 300-ms laser exposure per frame with a z-step of 0.5 µm between frames. Each time point consists of 43 z-steps with an average time interval of 14 s between time points. Shown is an inverted-grayscale 3D stack of the embryo. Timestamps represent minutes:seconds. The video starts in the middle of the embryonic two-cell stage (00:14) and ends at the beginning of the four-cell stage (13:46) to show the localization of klp-7 to the centrosomes, microtubules, and chromatin during mitosis. The bounding box (dashed black lines) represents 60 × 35 × 19 µm.

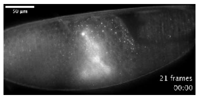

Video 4. **Maximal-intensity projection of a *D. melanogaster* embryo expressing an exogenous Axin-EGFP construct.** Images were acquired by using a 40× 1.3-NA oil-immersion objective, with the embryo mounted in a poly-L-lysine-coated chamber A. Each frame was acquired with a 300-ms laser exposure, a z-step of 0.5 µm, and a total z-range of 11.5 µm. The average time between time points is 7.94 s. The timestamp (lower right) is in the format of minutes:seconds. Image astigmatism in this video is a characteristic of the objective, which does not have flat-field correction to increase the number of photons transmitted through the objective.

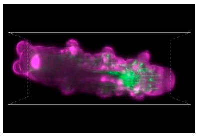

Video 5. **3D projection of a fixed and stained *H. dujardini* adult.** Actin fibers (green) are stained by using Oregon Green–tagged phalloidin, and the cuticle (magenta) is stained by using WGA. Images were acquired by using a 60× 1.2-NA water-immersion objective, with the adult tardigrade mounted in chamber A. Each frame was acquired with a 100-ms laser exposure, a z-step of 0.5 µm, and a total z-range of 80.5 µm.

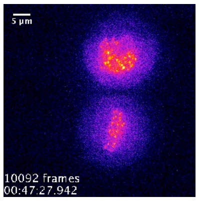

Video 6. **Maximal-intensity projection of two mitotic HeLa cells stably expressing Hec1-EGFP to mark mitotic kinetochores.** Images were acquired by using a 60× 1.49-NA oil-immersion objective with the cells mounted in chamber B. Each frame was acquired with a 200-ms laser exposure with a z-step of 0.3 µm between frames, with a total z-range of 17.4 µm. Shown is a 2D maximal projection of the stack with a "Fire" false coloration (Fiji). The timestamp (lower left) represents hours:minutes:seconds.milliseconds since acquisition began, as well as the cumulative number of frames acquired. The video is started after 10,000 frames to illustrate anaphase separation of kinetochores and subsequent reduction of Hec1-EGFP from the kinetochore from the lower cell. The upper cell is in prometaphase and serves as a visual photobleaching control.

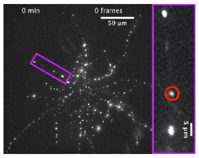

Video 7. **Maximal-intensity projection (after deconvolution, eight rounds of Richardson-Lucy algorithm) of an *A. gossypii* cell expressing an exogenous fluorescent histone construct, H4-GFP, to visualize nuclear movement and divisions through time.** Time stamps for the images are shown in the upper left, and the cumulative number of frames acquired is in the upper right. The purple region of interest in full-field image is rotated and magnified by 3.7× on the right and outlined in purple. Nuclei of interest are encircled in colors, corresponding to colors in Fig. 6. The birth of new nuclei at mitoses are indicated with colored arrows.

Provided online are two tables in Excel and a ZIP file containing three supplemental data files.

Table S1. Worksheet (programmed by using Microsoft Excel) containing Eqs. 1, 4, 5, 6, 7, and 9 from Materials and methods. This worksheet can accept inputs describing the users' existing microscope system, and in return the worksheet will automatically calculate ideal parameters for constructing a LITE system around the user's microscope. The following are descriptions of the four steps for using Table S1: (1) Select the tab titled "USE THIS TAB." Enter the magnification (M), numerical aperture (NA), field number (FN), and immersion medium for the desired detection objective. (2) Select from the drop-down list the desired fluorophore. If the desired fluorophore is not reported, change tabs to "Fluors" and enter excitation/emission information on that tab. Return to "USE THIS TAB," and the new fluorophore should appear. (3) Decide if the provided fluorophore excitation wavelength (cell B11) matches the wavelength available in the user's system. If no, enter the closest available wavelength in nanometers. (4) Decide if the full objective FOV (cell B14) is desired for imaging. If no, enter the desired FOV length in

micrometers. The worksheet will then output several parameters in the "Outputs" section. The most useful of these outputs are bolded and should be considered when constructing a LITE system according to the assembly instructions presented in Table S2.

Table S2. Worksheet (programmed by using Microsoft Excel) containing a list of parts and assembly instructions for the LITE system. A complete list of parts can be found under the "PARTS" tab, including names, descriptions, and model numbers/hyperlinks. Letter/color descriptions correspond to the visual assembly diagram available in the Data S2 file. A step-by-step assembly procedure can be found under the "ASSEMBLY" tab.

Data S1. CAD file (AutoCAD) containing scaled drawings of all LITE system parts. All units are in millimeters. Data S1 file contains parts A–U (Table S2) that are labelled and colored according to the parts list. All custom parts (J–O) are drawn to scale in the Data S1 file and can be used for manufacturing.

Data S2. Visual assembly instructions for the LITE system. All part drawings were exported from AutoCAD (Data S1 file). All parts are labelled (in colors and letters) according to the parts list (Table S2). Data S2 is a visual accompaniment to the assembly instructions included in Table S2.

Data S3. Two alternative methods for a multicolor LITE system. (A) Visual representation of a sample objective FOV (white box) and corresponding optimized tilted light sheet (cyan box). Sheet dimensions are listed in cyan. The lower half of (A) has been stretched vertically (100×) and compressed horizontally (0.5×) to assist in visualization. To use LITE with multiwavelength acquisition, we propose two alternative methods: (1) Fixed-angle/photomask and (2) variable-angle/photomask. (B) Method 1 of multicolor LITE is logistically simpler but optically less optimized. Method 1 requires selecting a preferred wavelength (shown is 488-nm excitation for EGFP) and keeping the tilt angle and photomask constant between different excitation wavelengths. The red and purple boxes are representations of light sheets generated around the FOV (white box) when the angle and photomask are kept constant for excitation wavelengths of 646 nm (JF646) and 383 nm (EBFP), respectively. Note that in method 1 shorter wavelengths (purple box) tend to slightly miss the far corners of the FOV, and longer wavelengths (red box) tend to over-illuminate outside the FOV. Over the visible electromagnetic spectrum, this effect does not prohibit imaging. (C) Method 2 of multicolor LITE is optically optimized but logistically complex. Method 2 requires changing the photomask dimensions and tilt angle for each excitation wavelength. Changing these two parameters is theoretically possible by replacing two components in the LITE system design: the adjustable angle plate and the photomask. The tilt angle can be controlled precisely in real time with an adjustable-angle piezoelectric motorized stage instead of the adjustable-angle plate. In addition, the photomask must also be modulated in real time. We propose modulating the photomask by replacing the static mask with a high-resolution liquid crystal display. With these part replacements, multicolor LITE can be optimized such that sheets of all wavelengths are more similar in w and L′. However, one drawback exclusive to method 2 is the inability to perform simultaneous multicolor excitation/acquisition, because each excitation wavelength requires a unique modulation to tilt angle and photomask dimensions.
(D) Rescaled images of light sheets from methods 1 and 2 for a variety of excitation wavelengths.

https://doi.org/10.1083/jcb.201710087
