# Supplementary figures and images for: LITE microscopy: Tilted light-sheet excitation of model organisms offers high resolution and low photobleaching
Source: J Cell Biol. 2018 May 7;217(5):1869–82. doi: 10.1083/jcb.201710087 (PMC5940309; doi:10.1083/jcb.201710087)

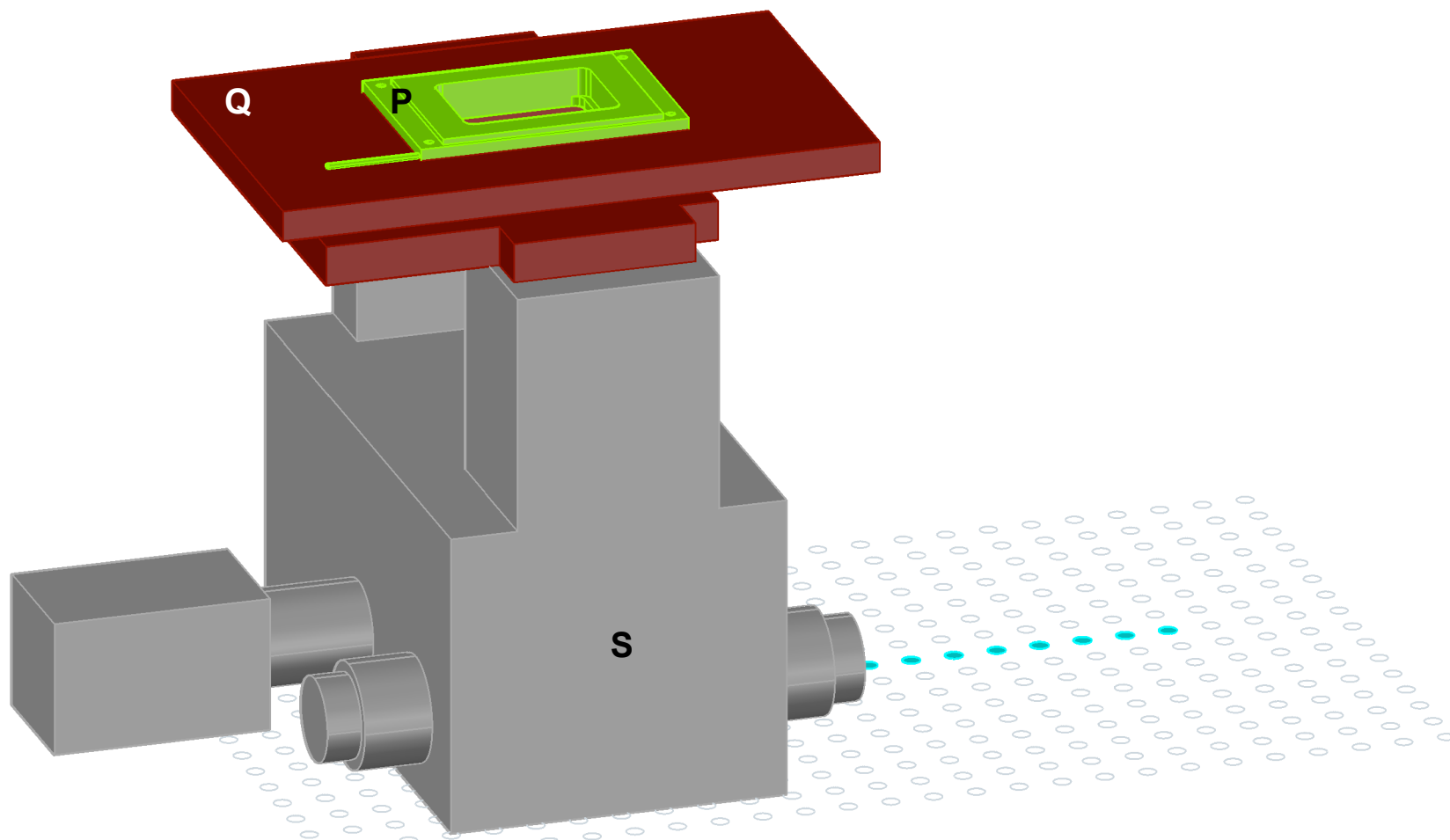

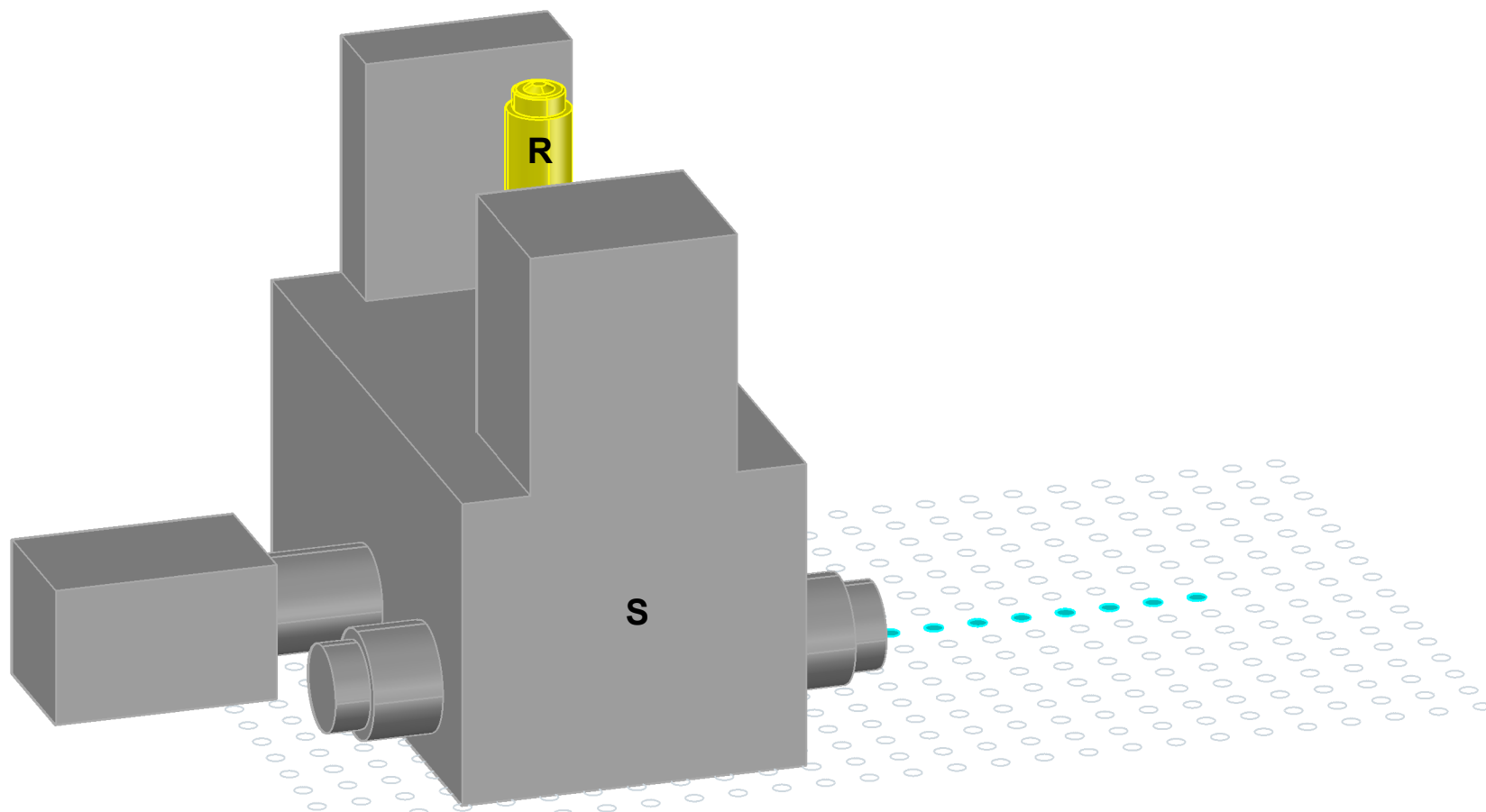

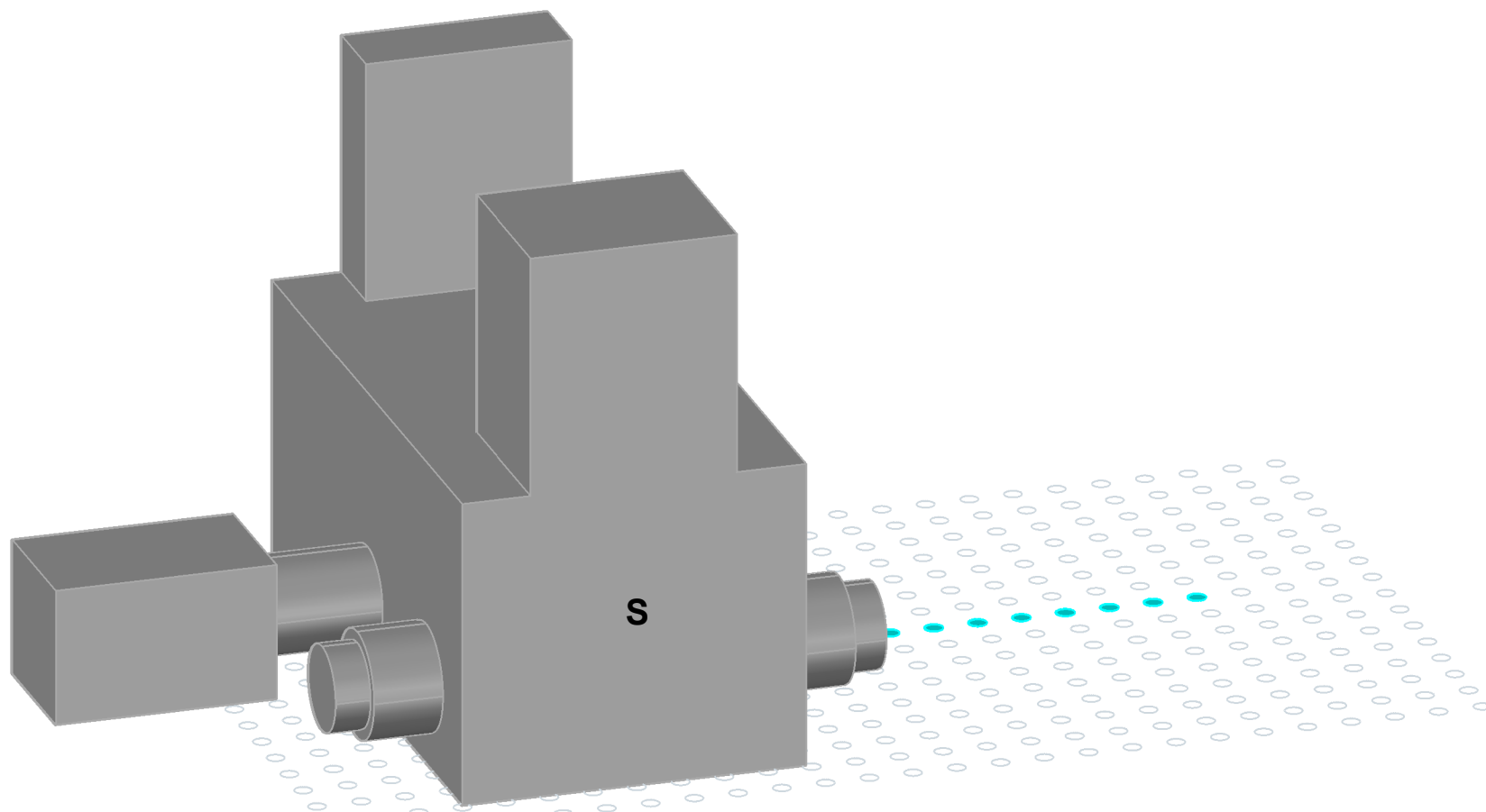

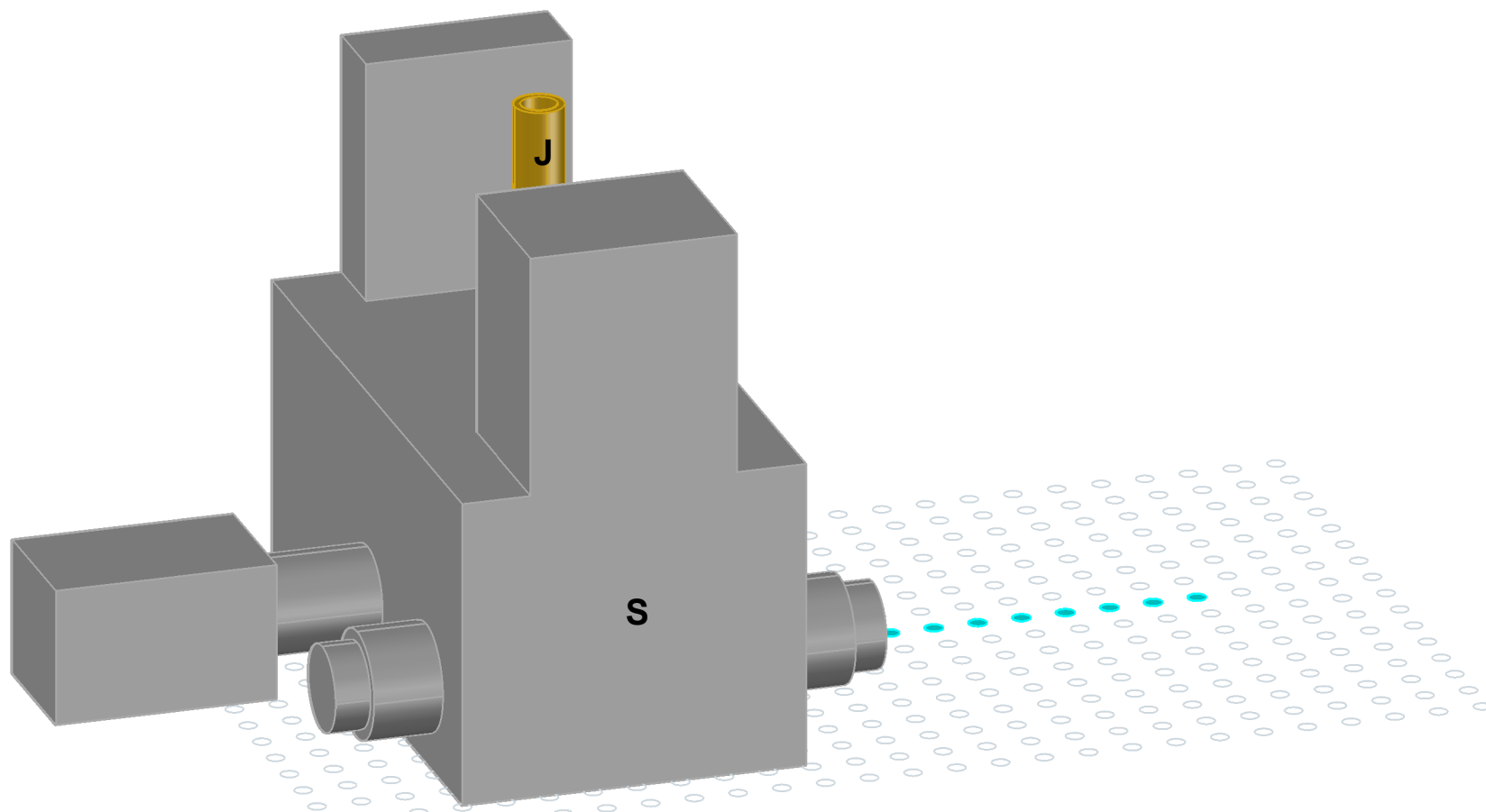

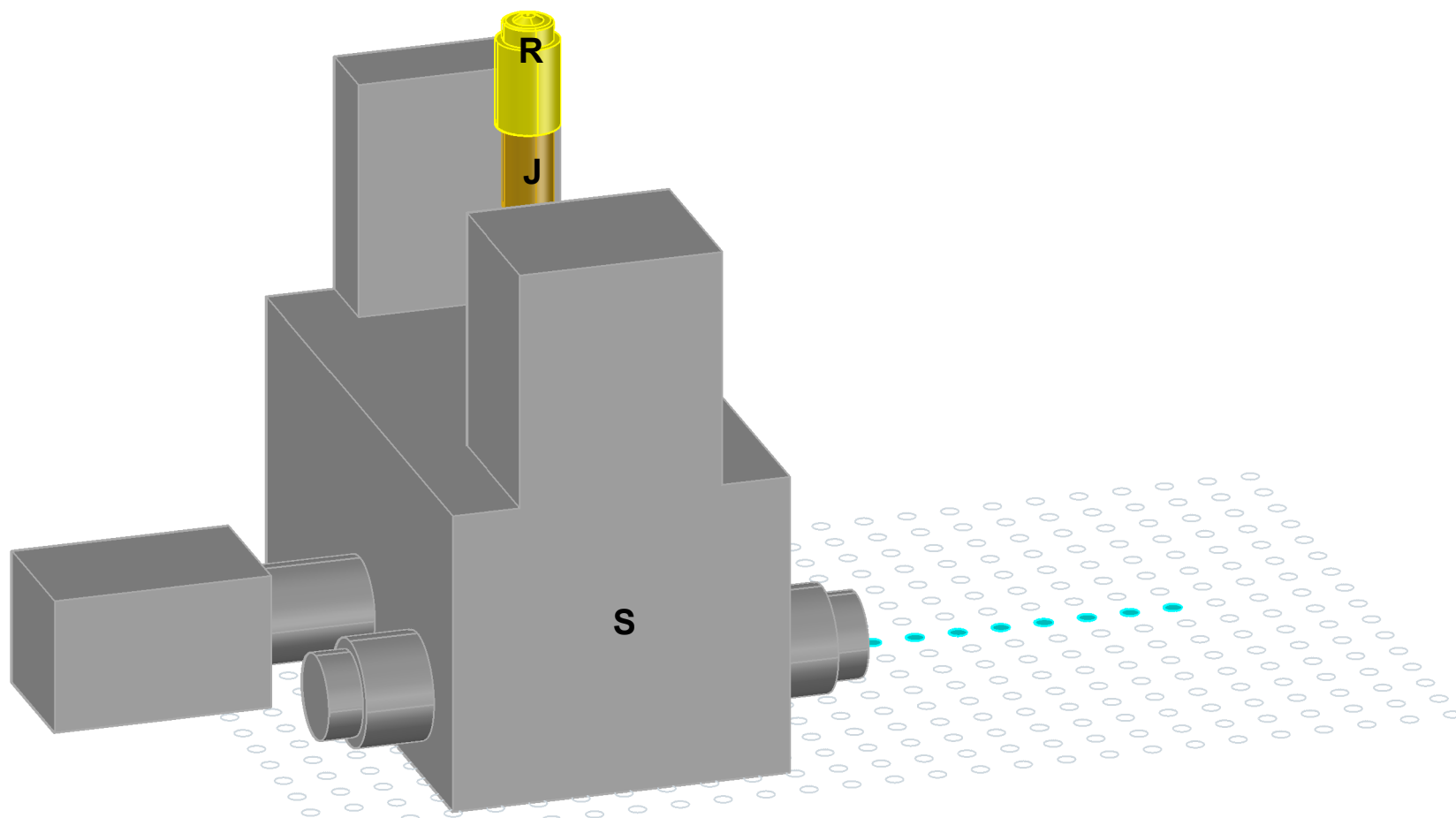

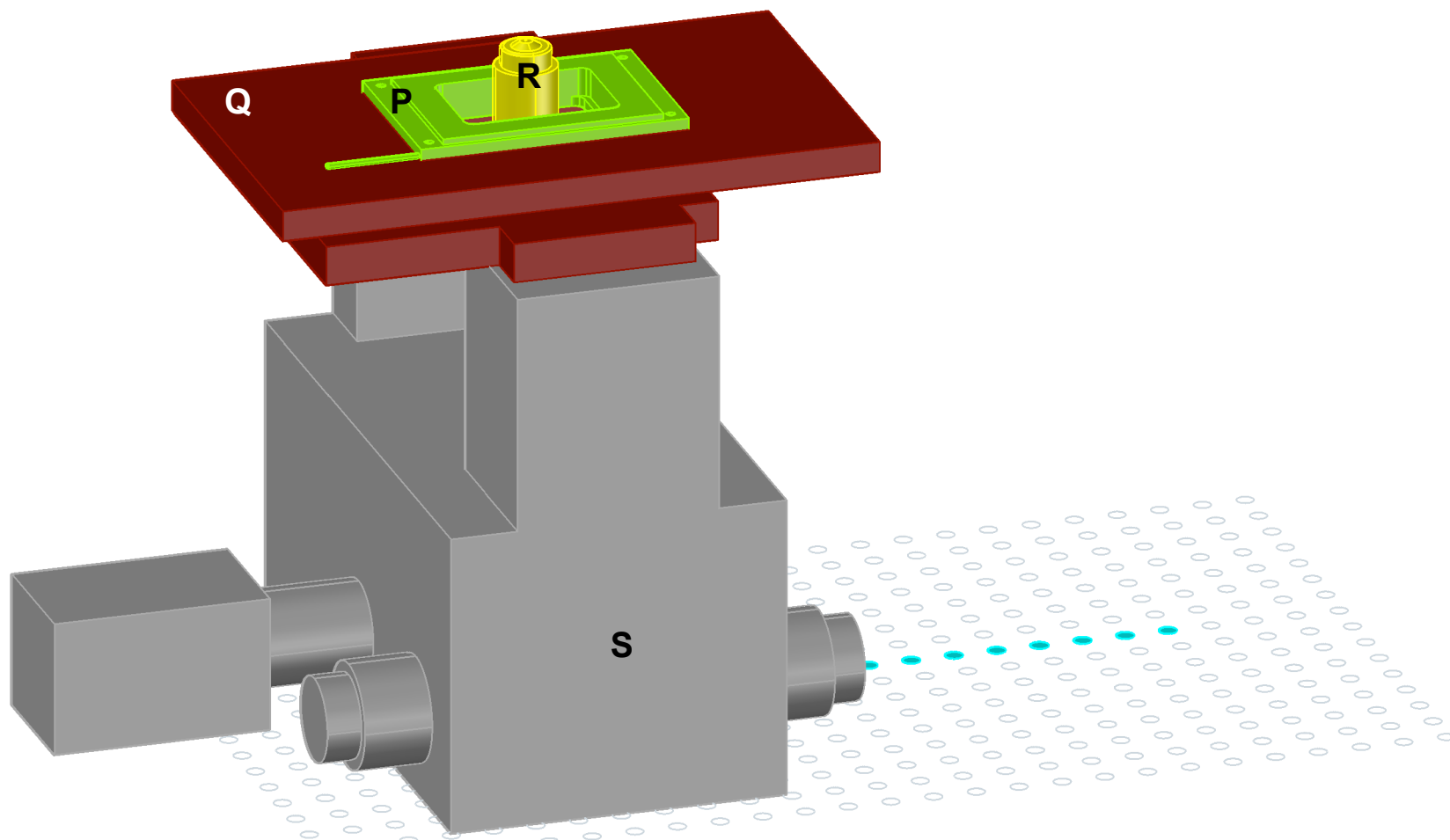

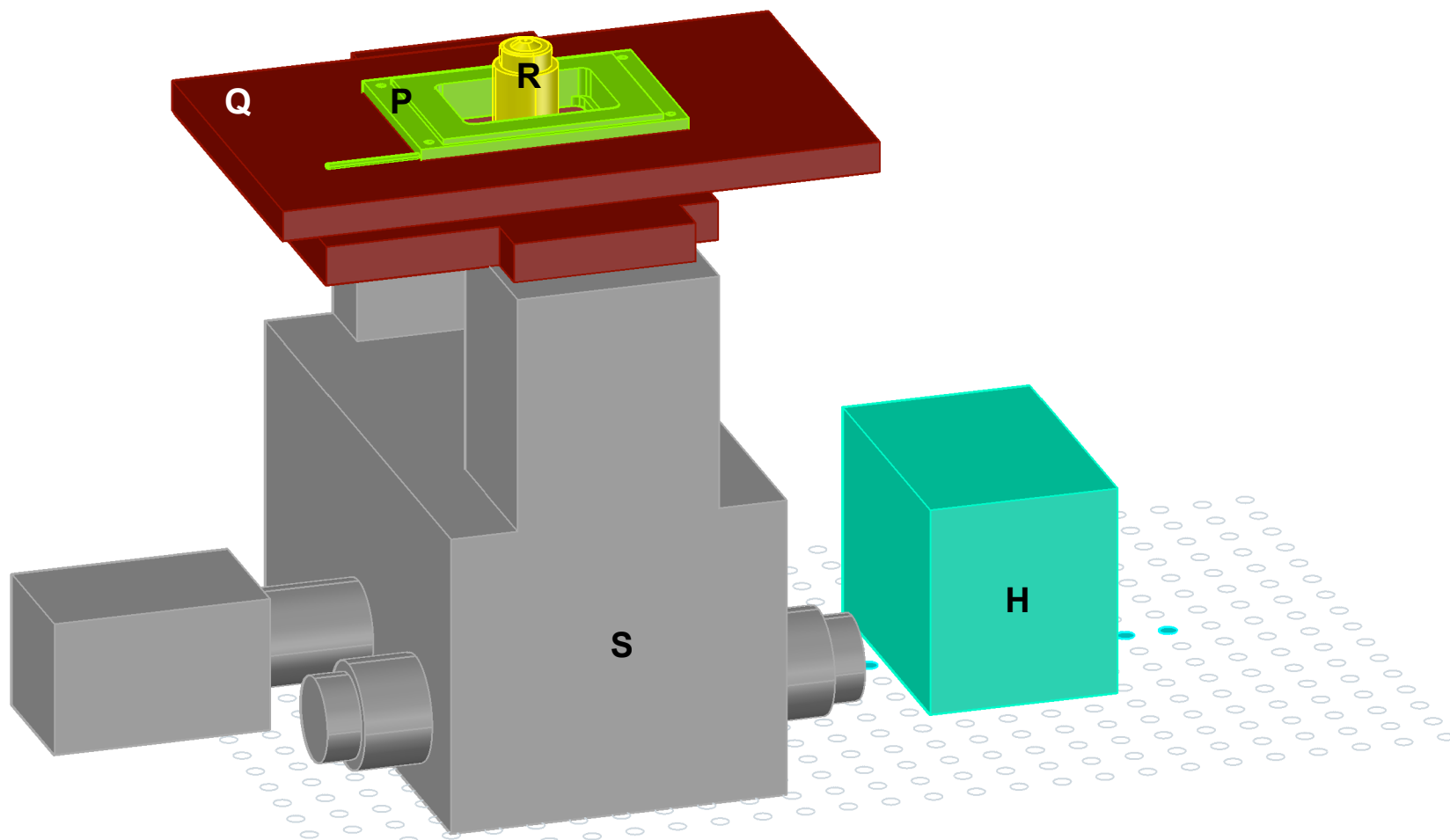

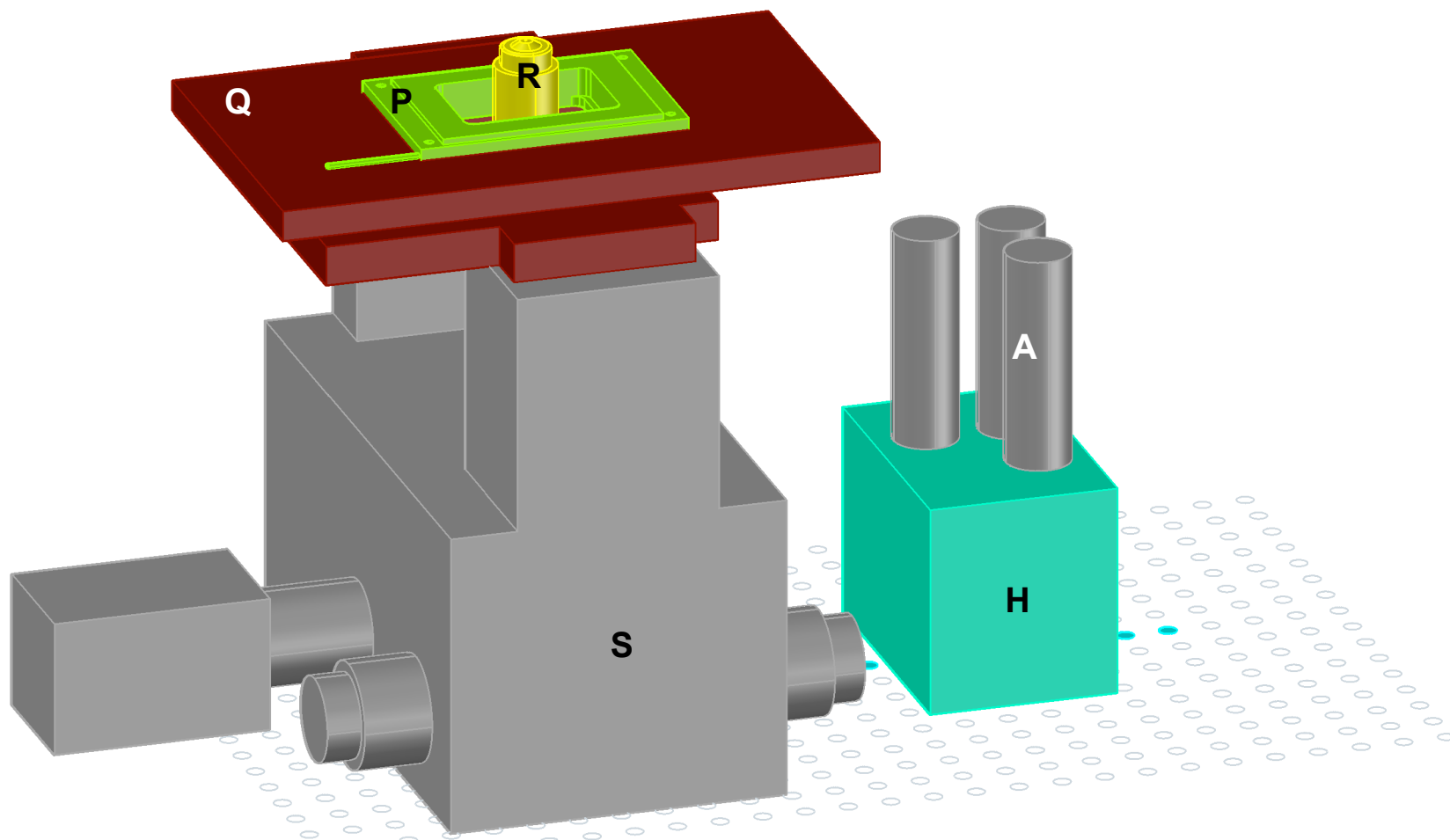

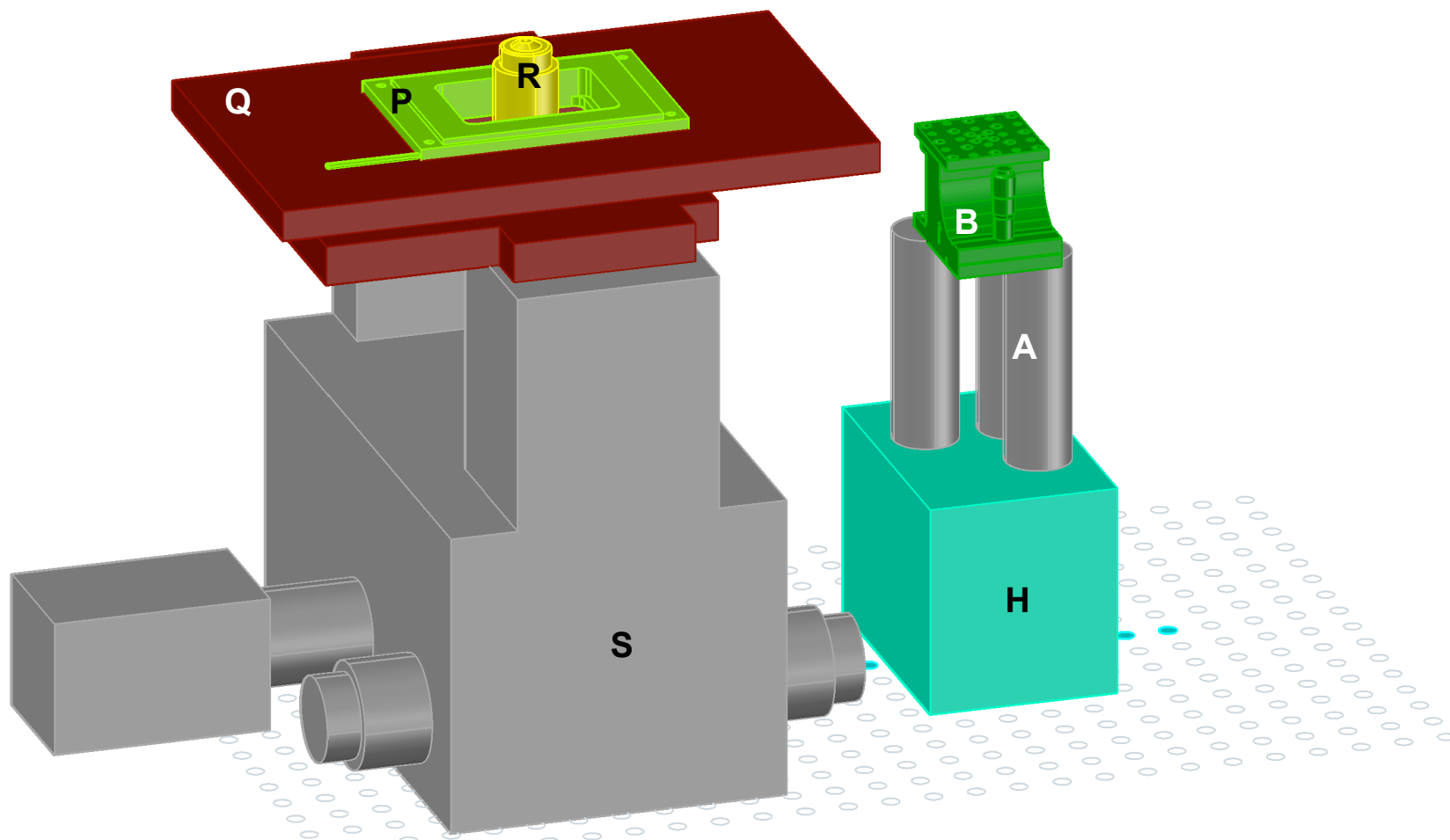

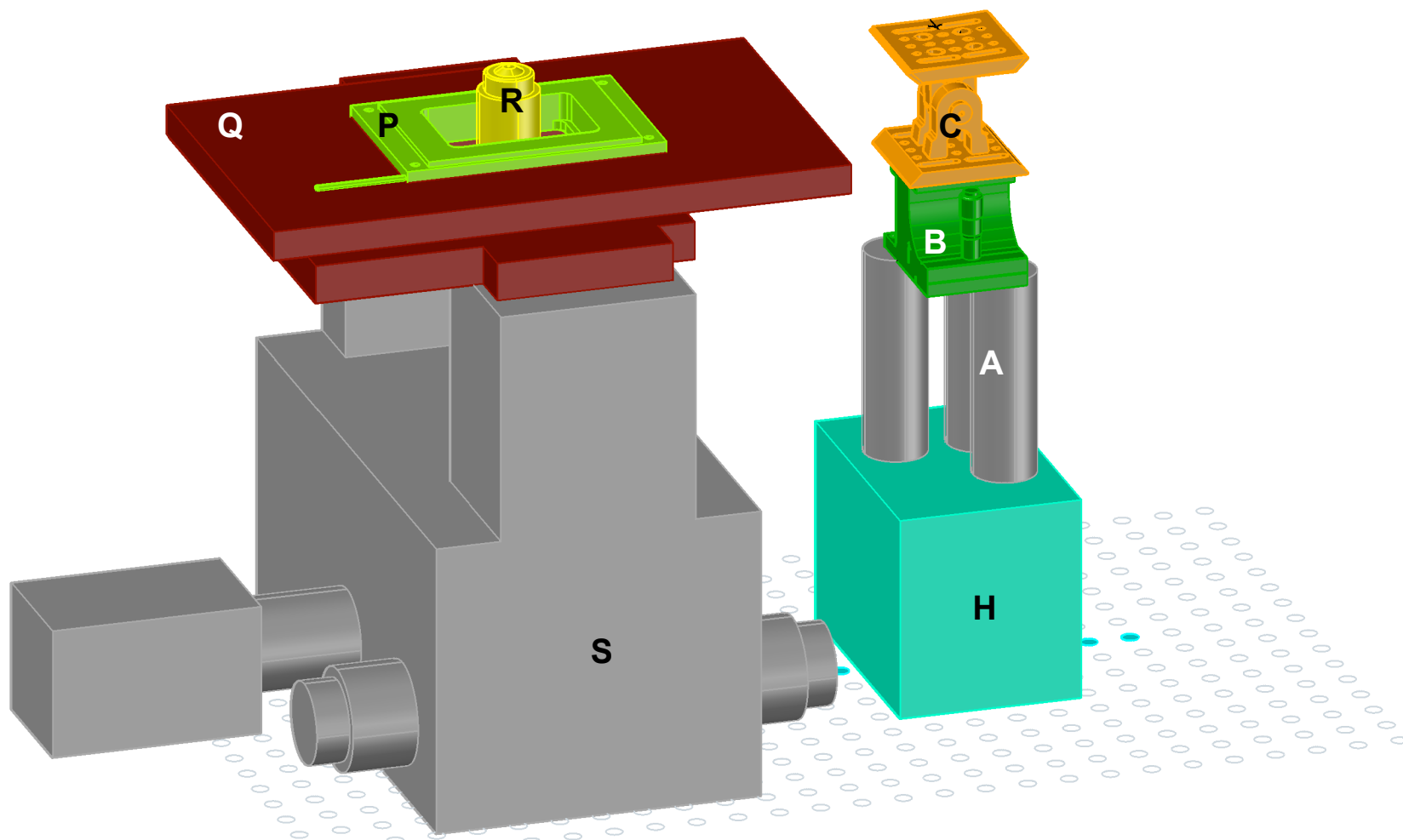

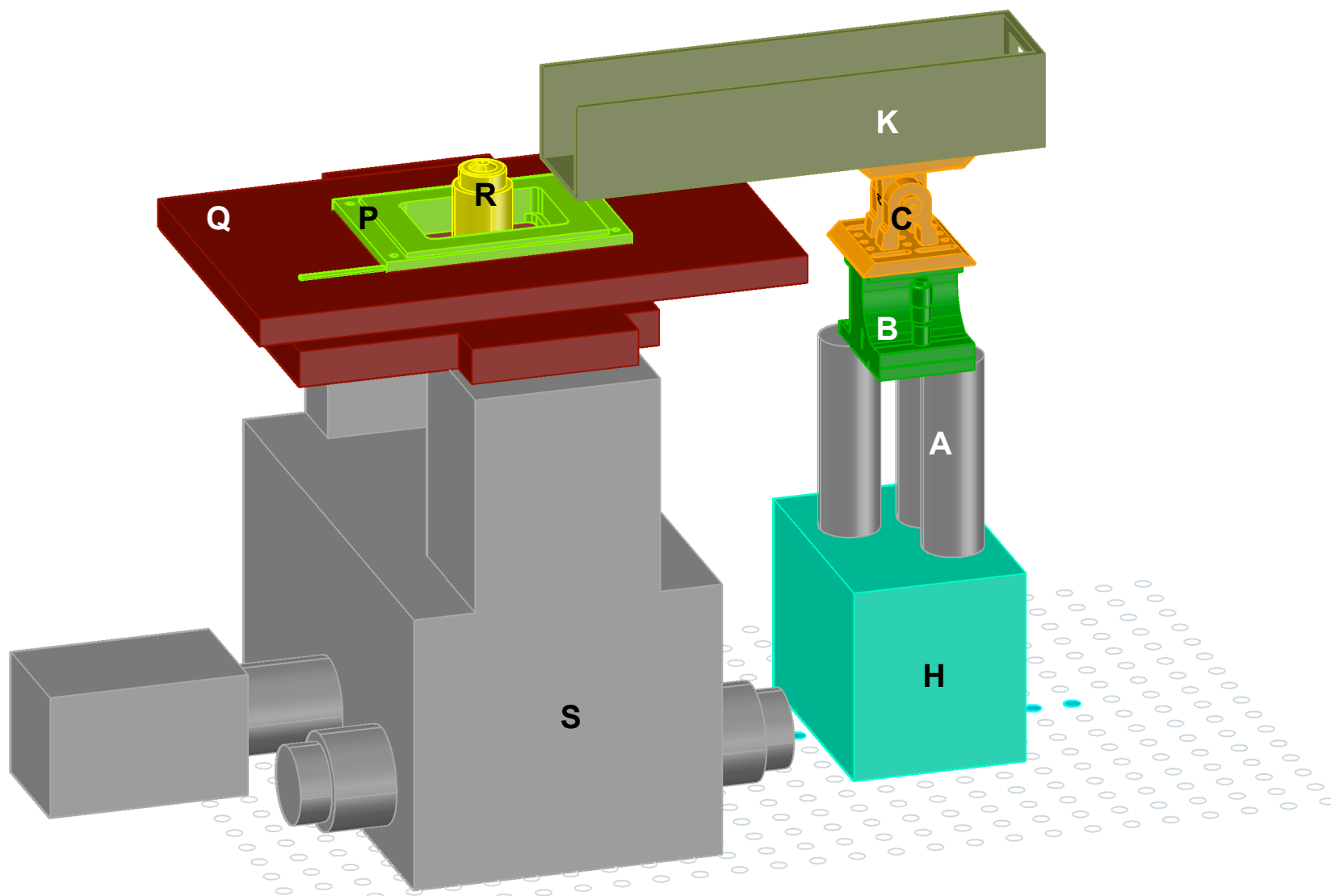

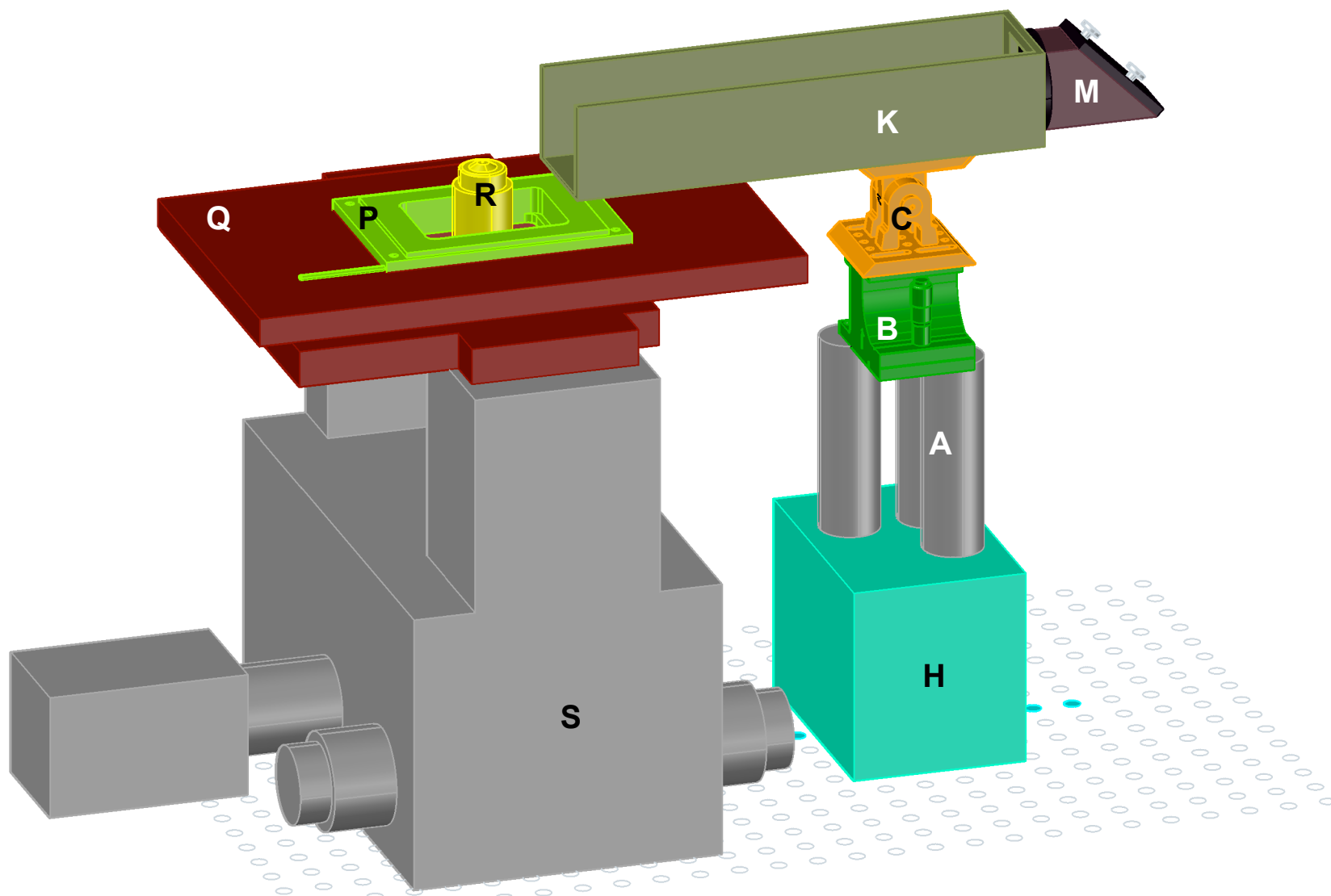

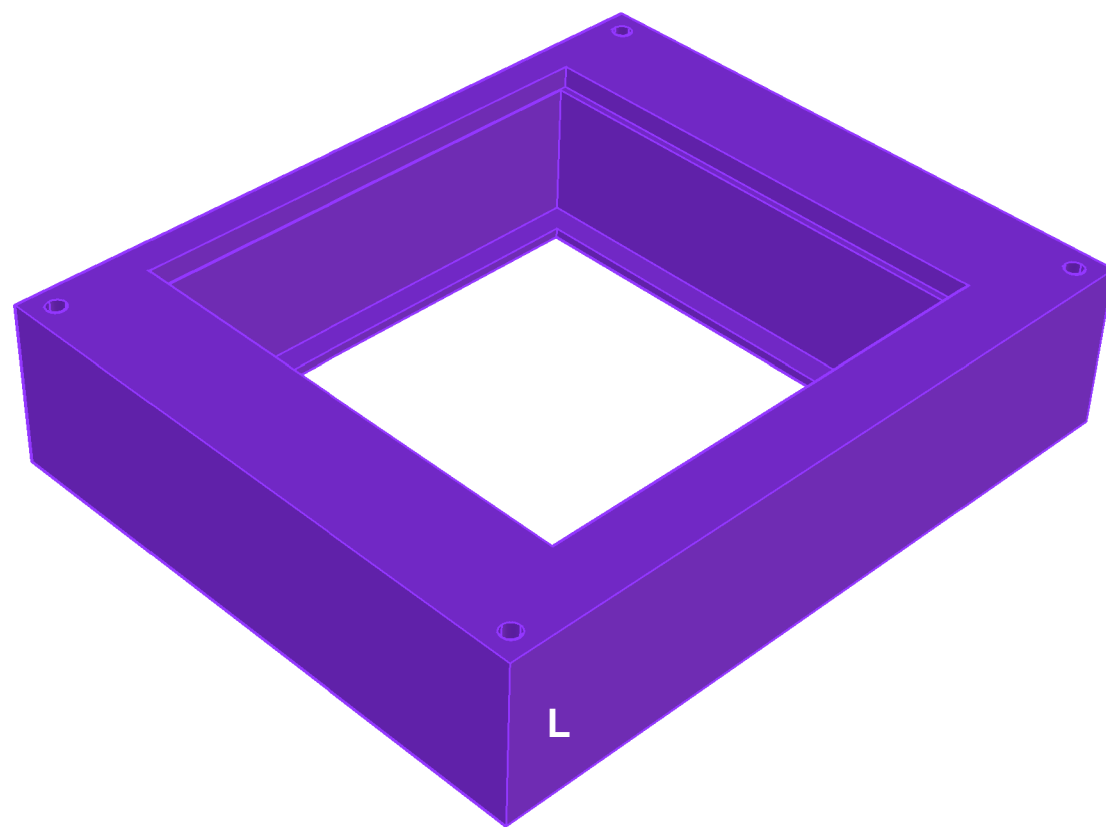

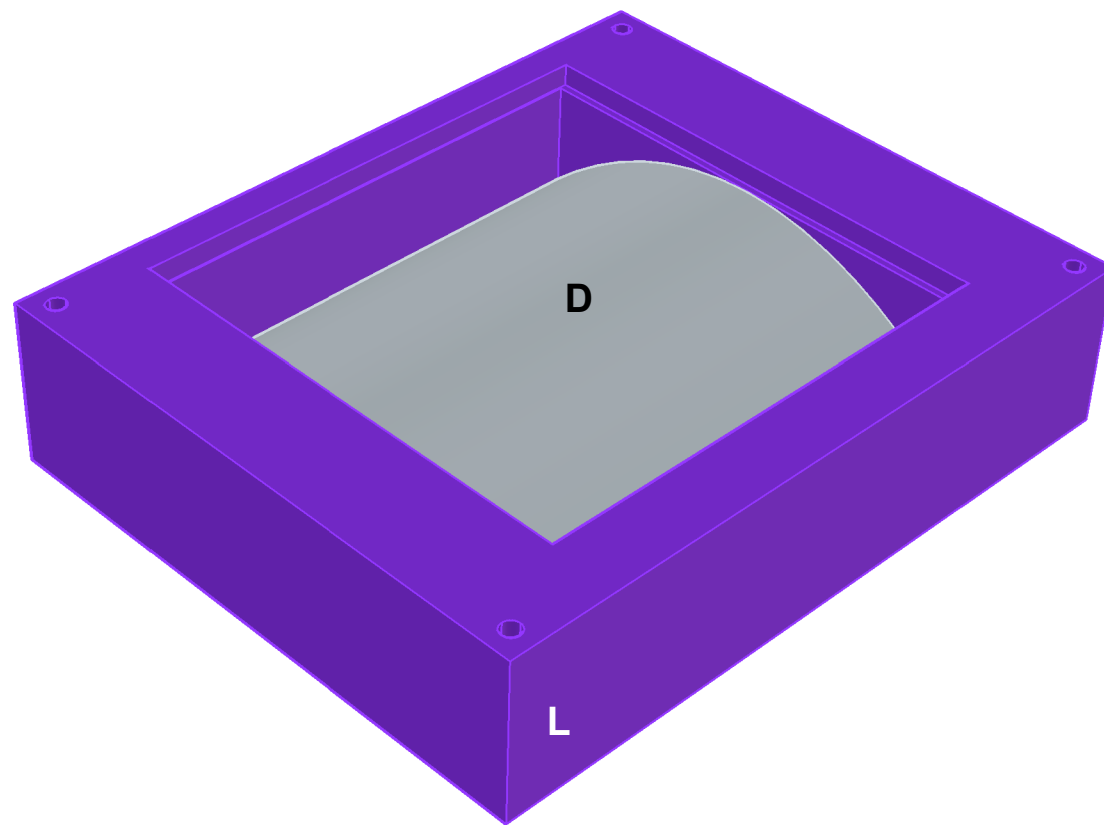

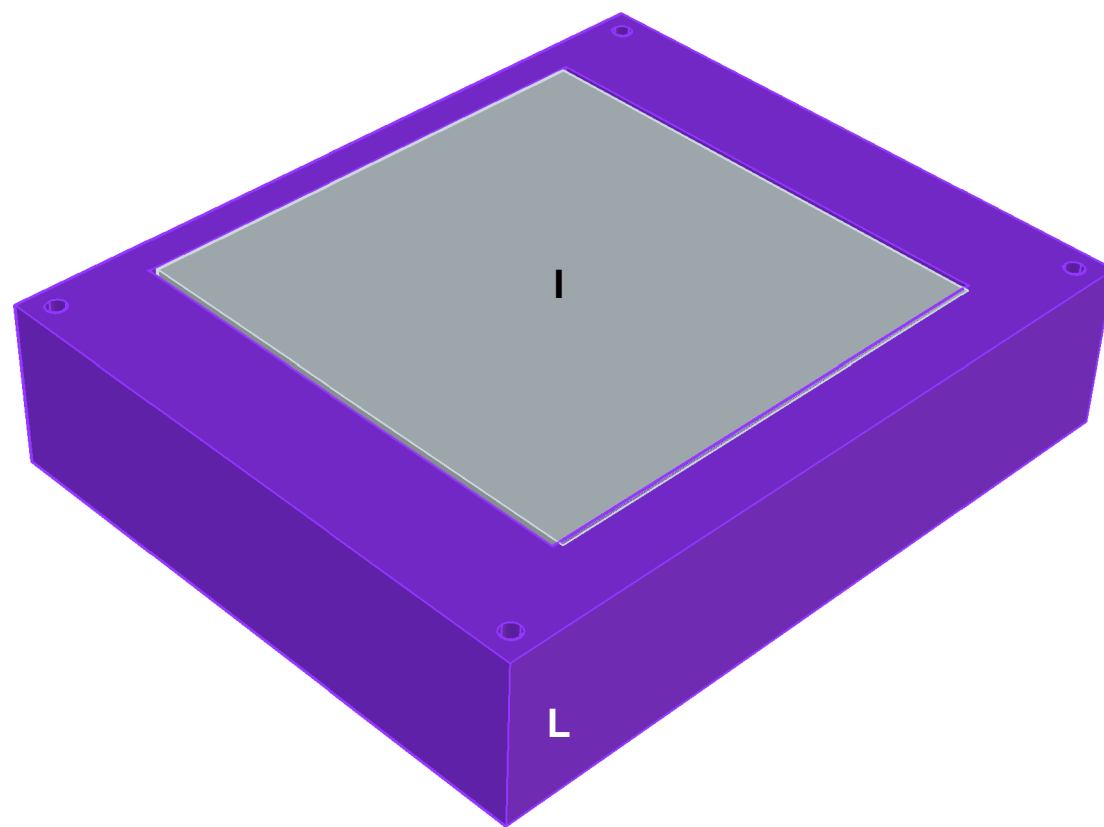

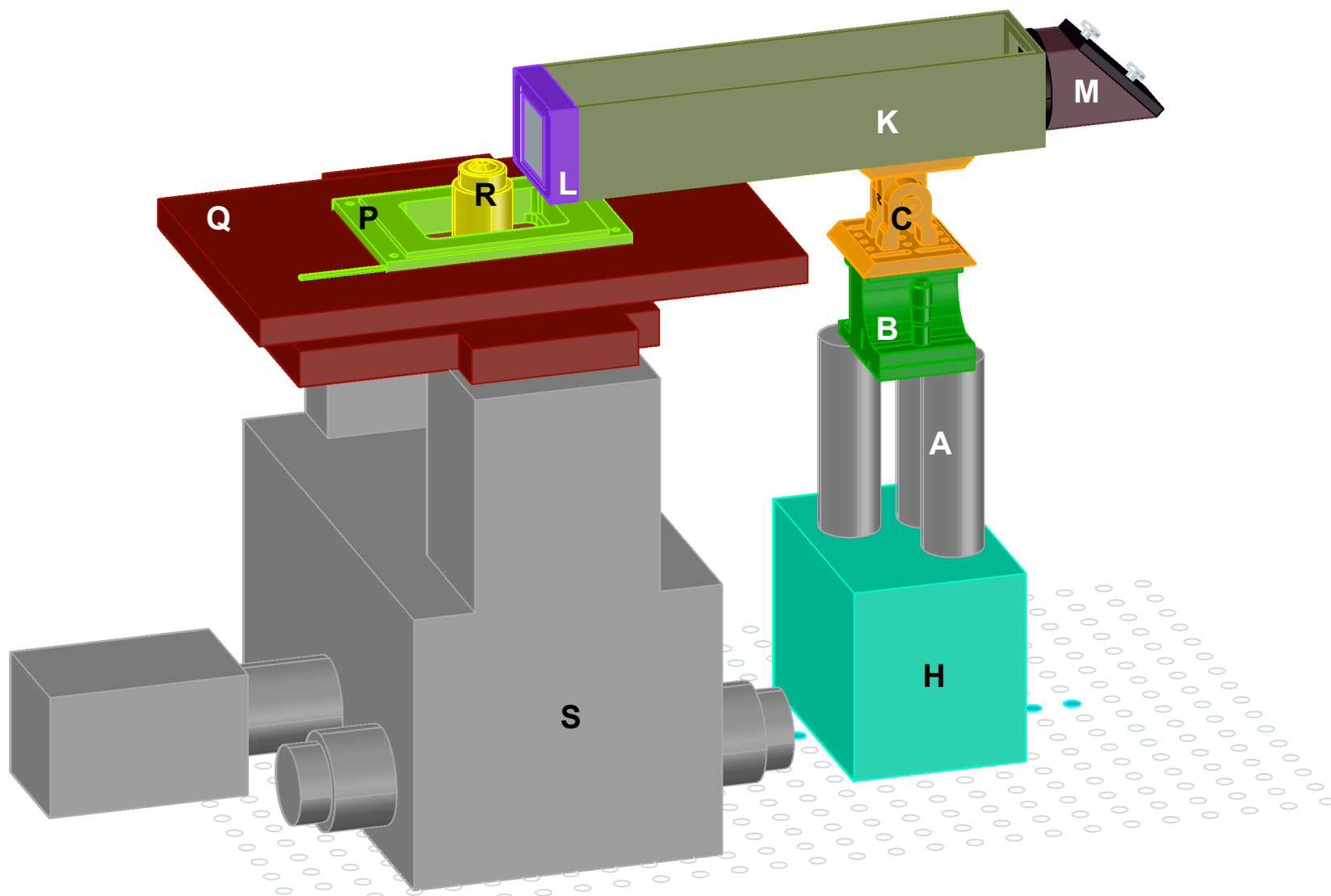

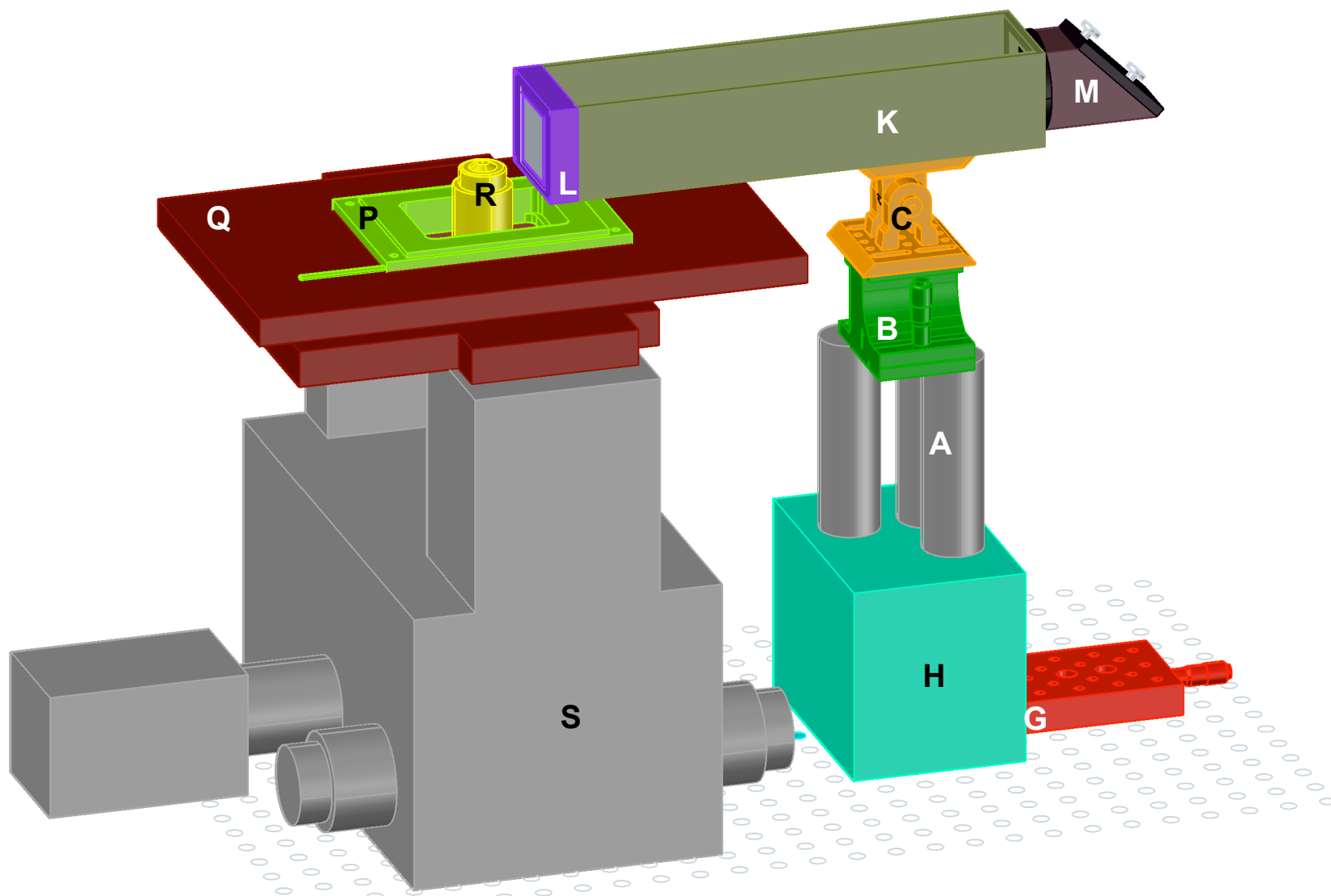

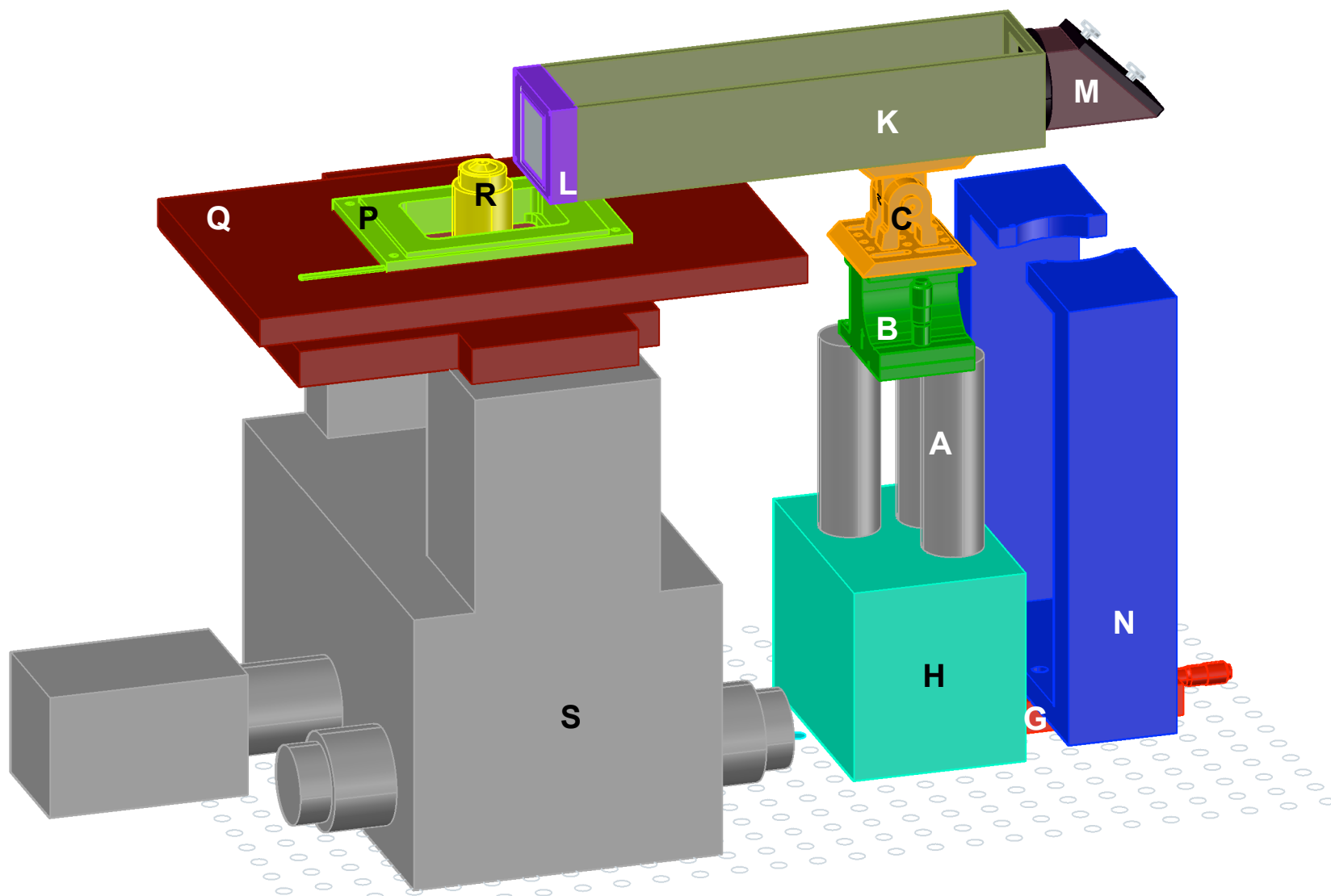

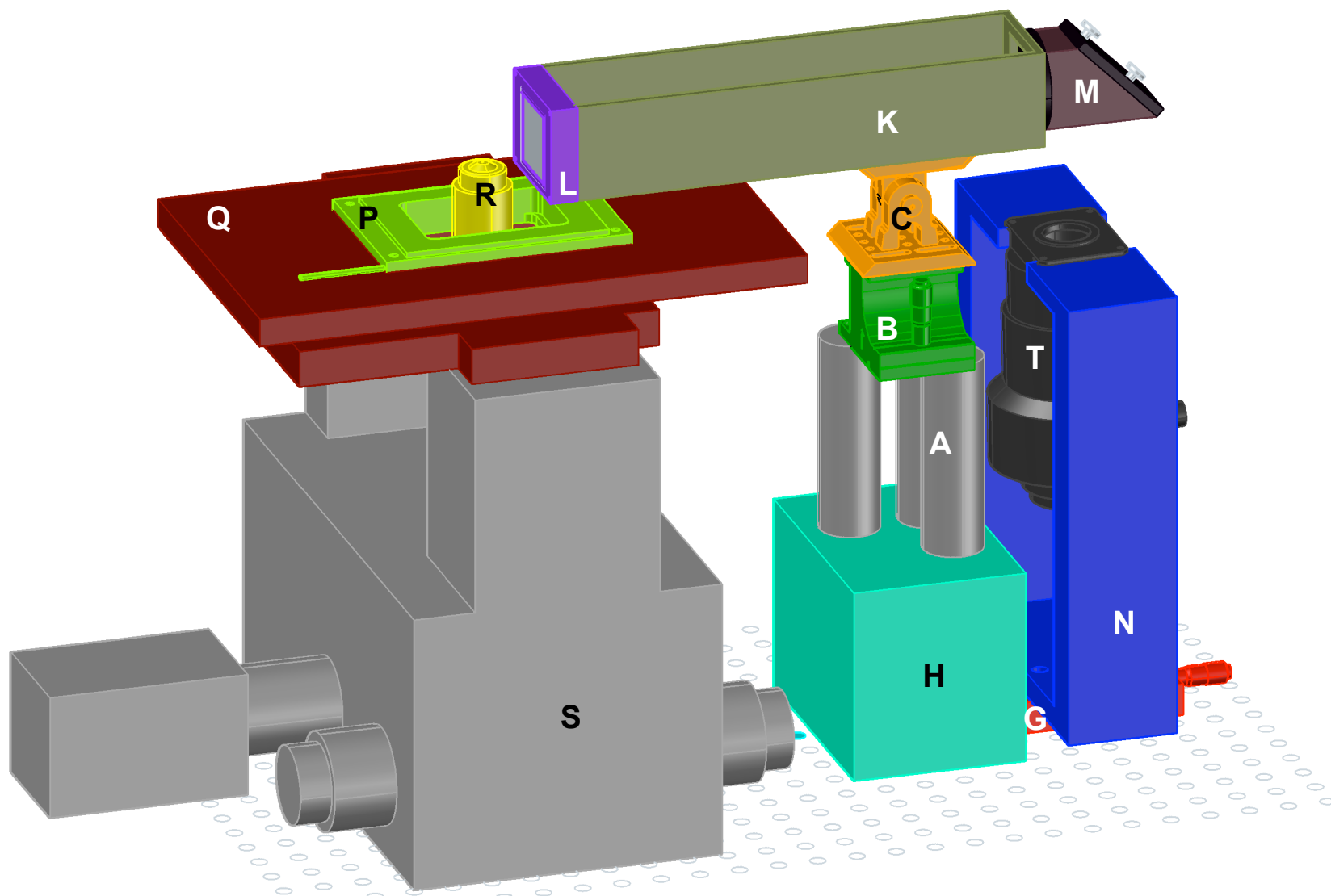

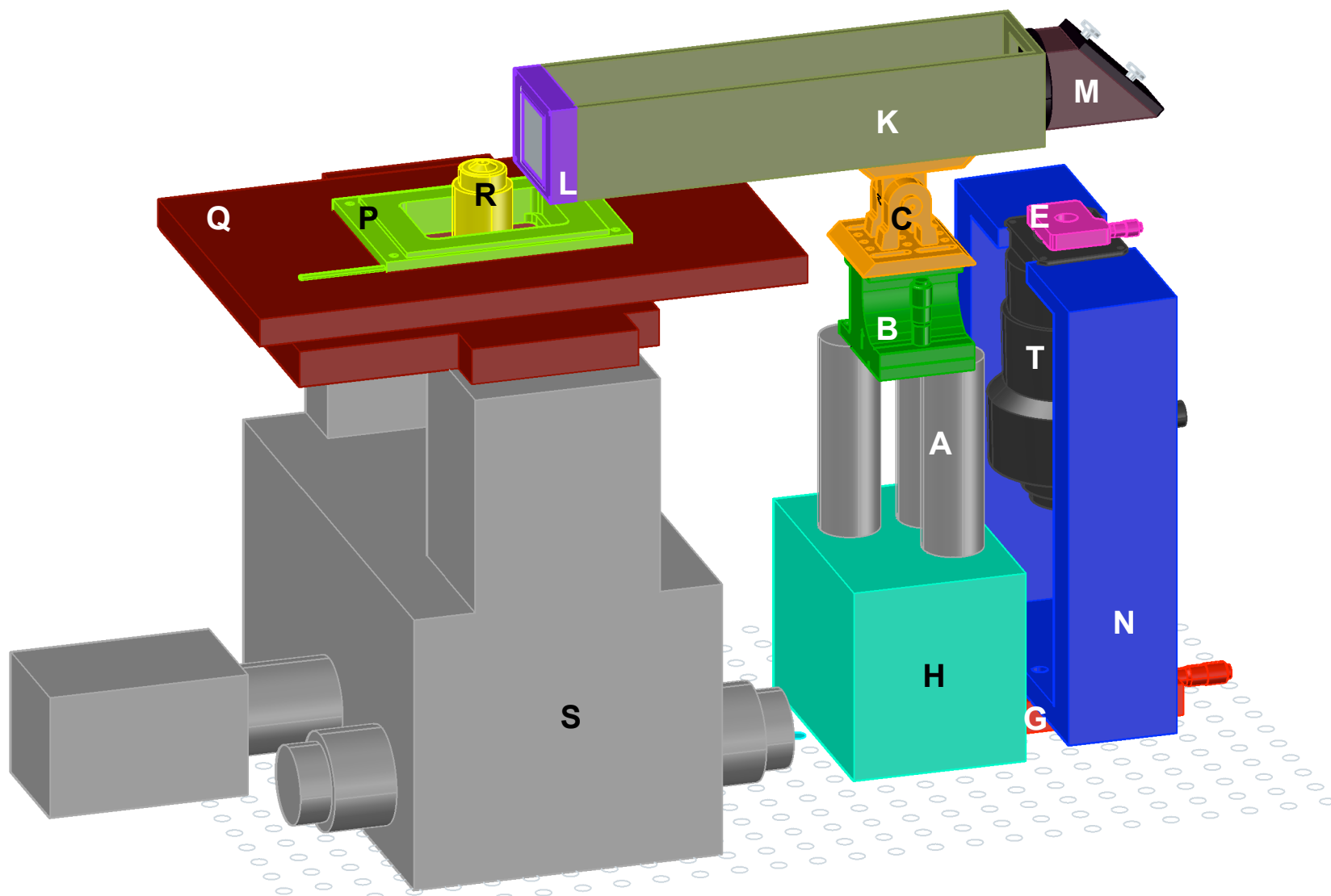

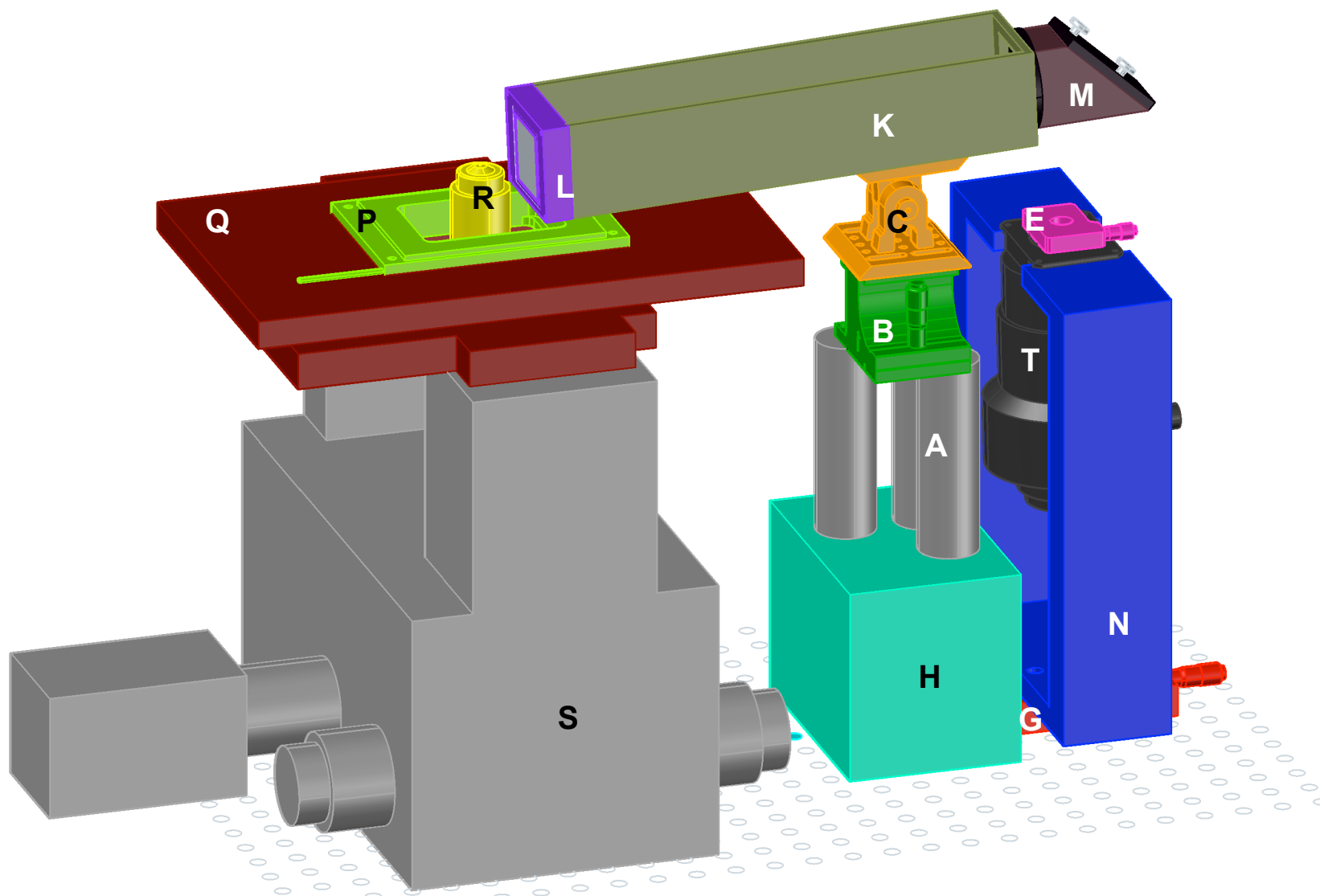

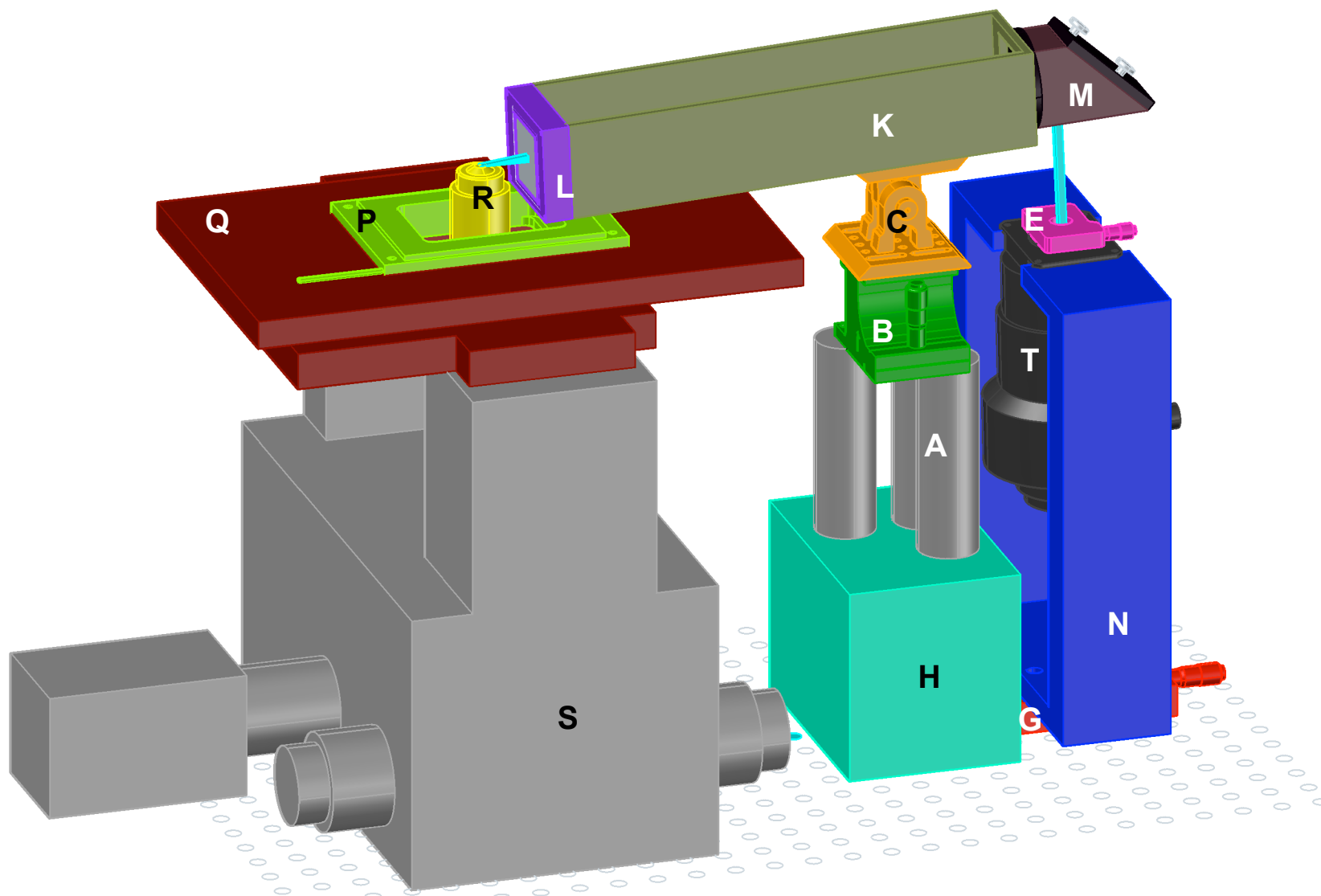

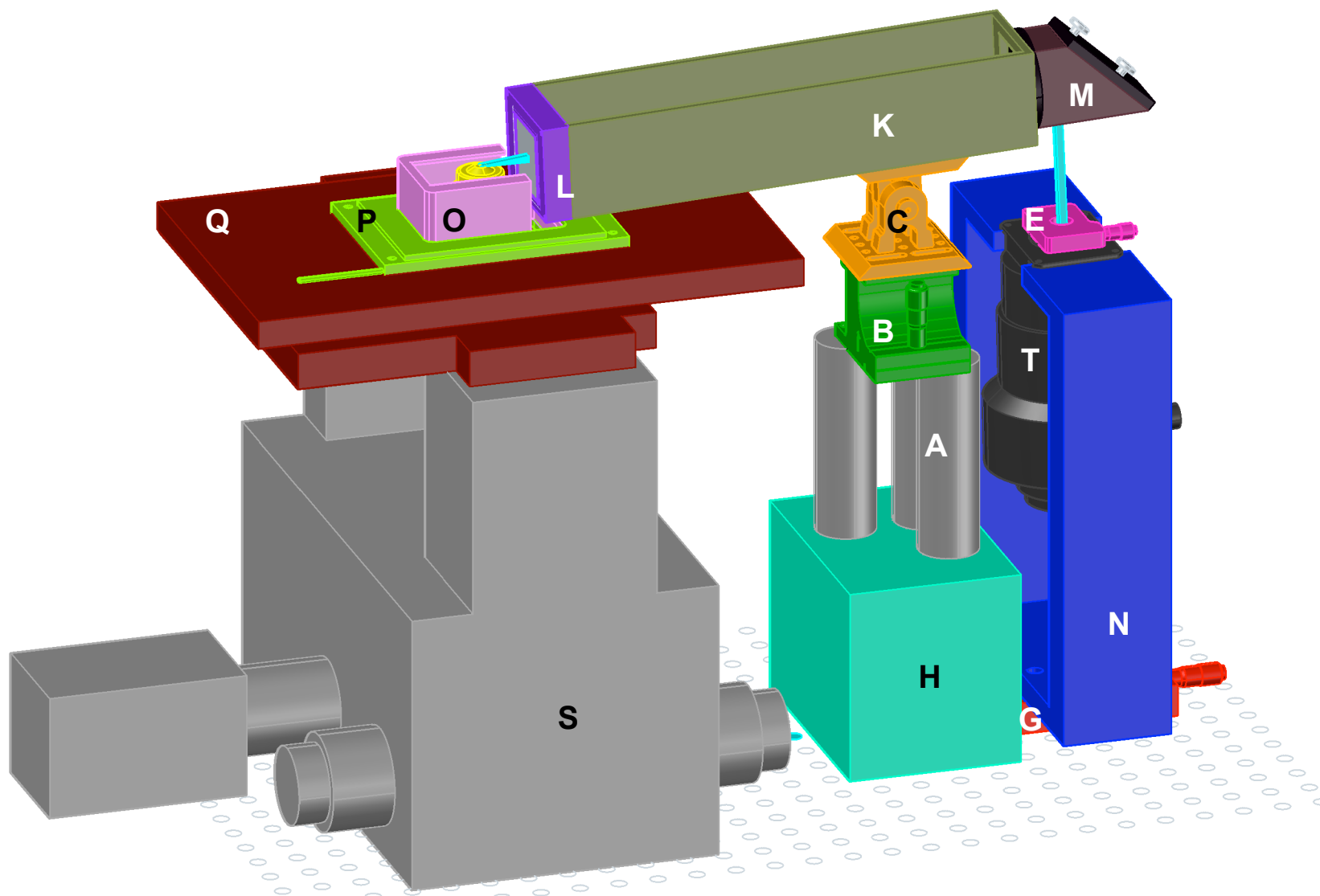

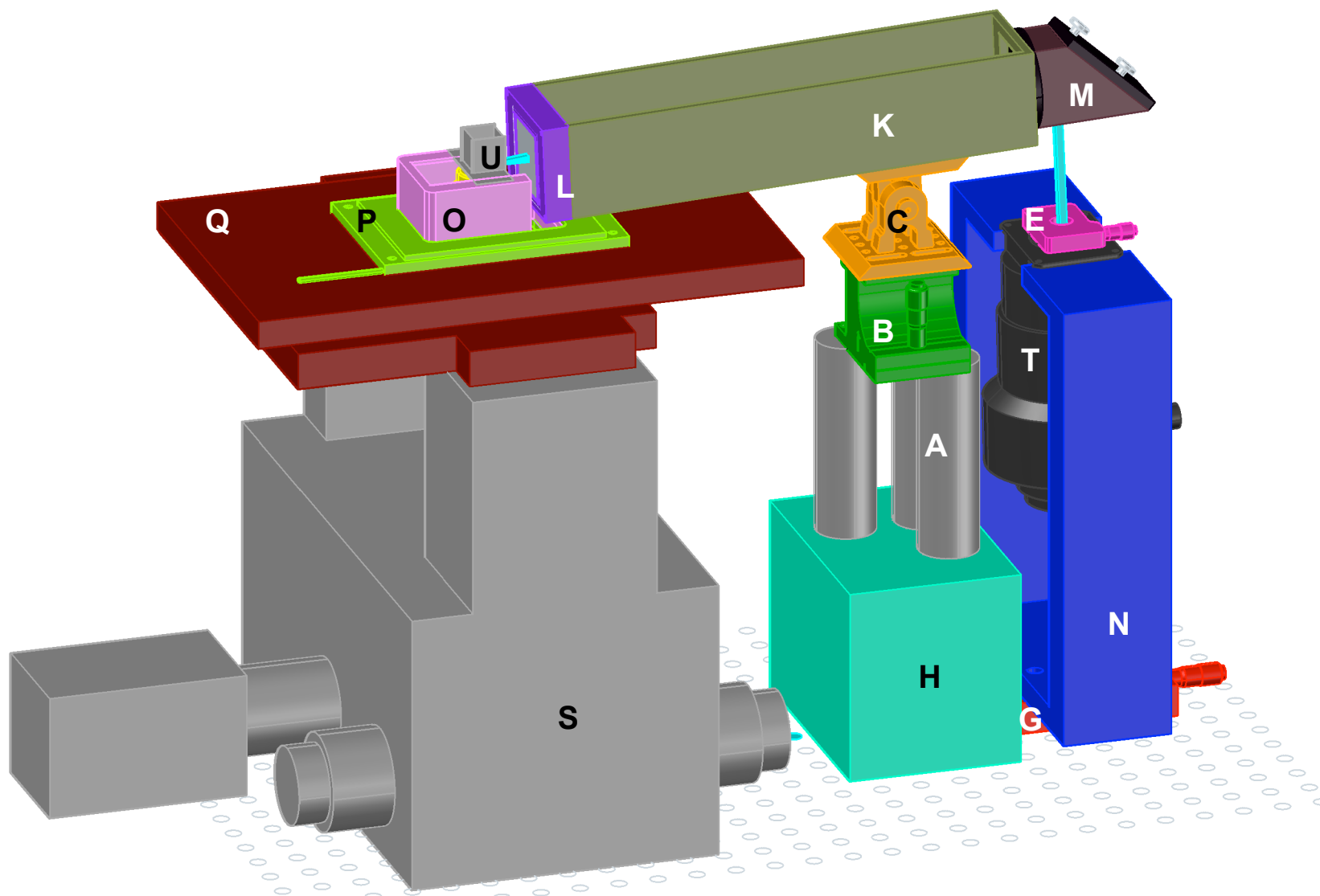

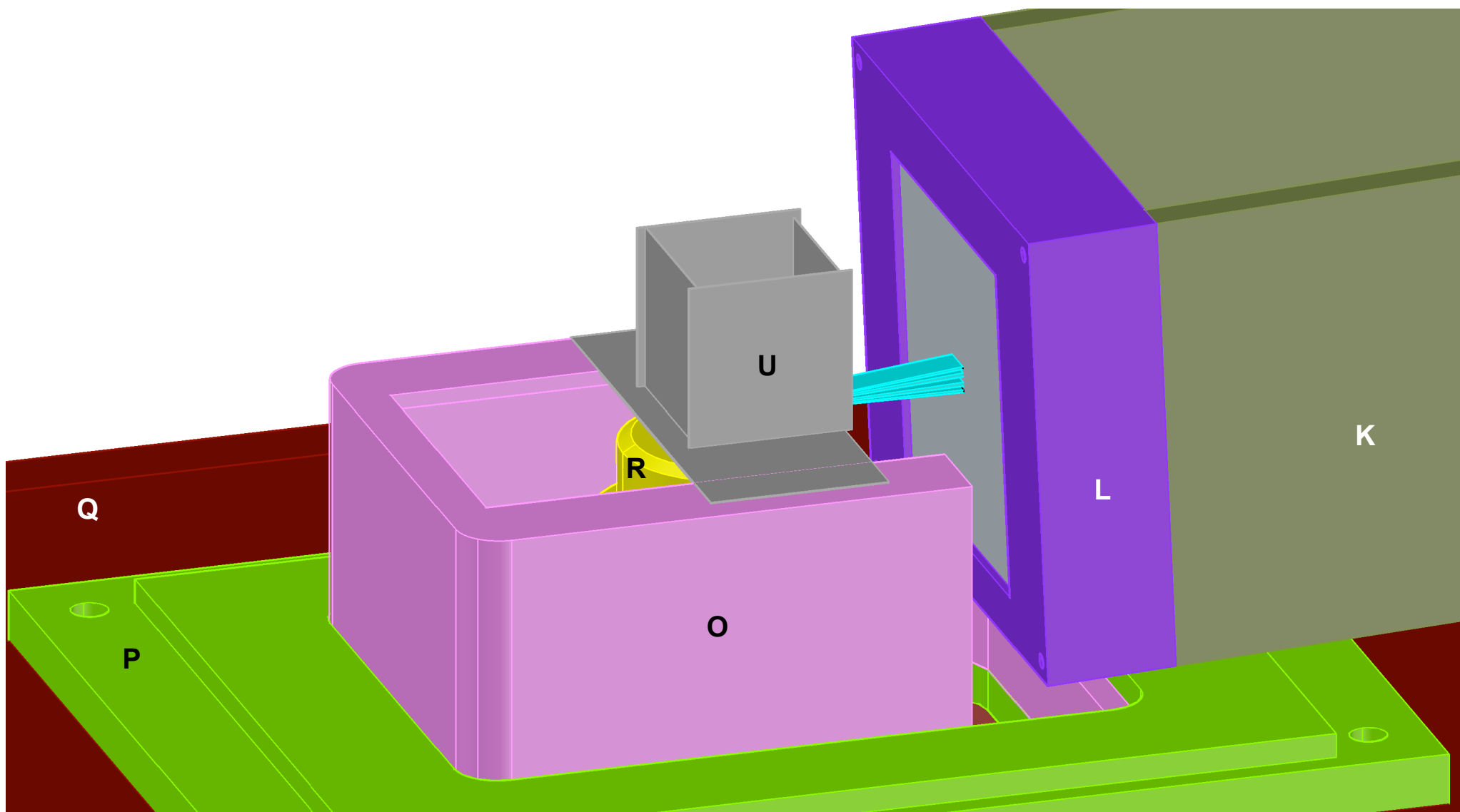

Supplement: Data S1-S3 (ZIP) [file JCB_201710087_DataS1toS3.zip › JCB_201710087_DataS2.pdf]

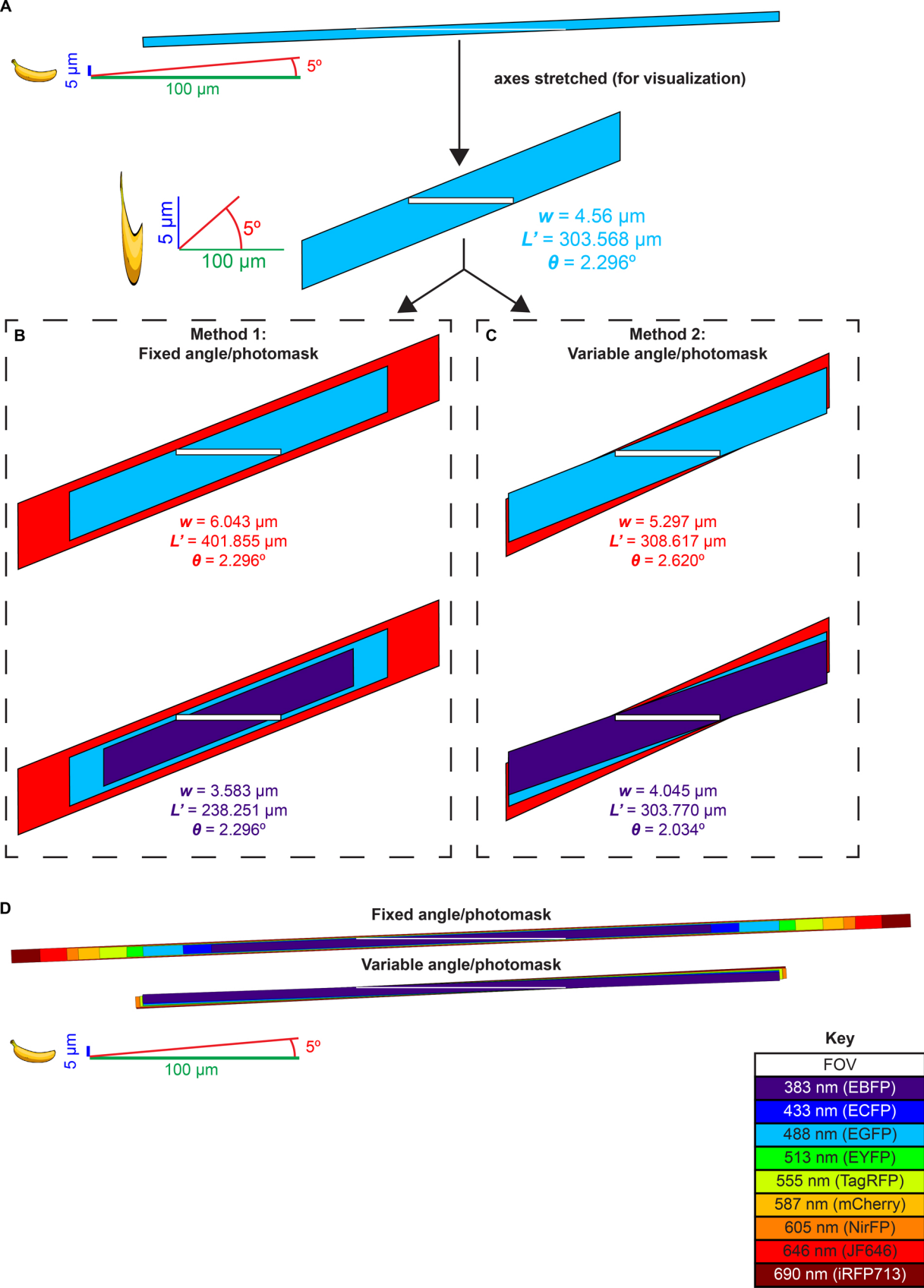

Supplement: Data S1-S3 (ZIP) [file JCB_201710087_DataS1toS3.zip › JCB_201710087_DataS3.pdf]
